# A genetic screen in Arabidopsis reveals the identical roles for RBP45d and PRP39a in 5’ cryptic splice site selection

**DOI:** 10.3389/fpls.2022.1086506

**Published:** 2022-12-22

**Authors:** Weihua Huang, Liqun Zhang, Yajuan Zhu, Jingli Chen, Yawen Zhu, Fengru Lin, Xiaomei Chen, Jirong Huang

**Affiliations:** ^1^ Shanghai Key Laboratory of Plant Molecular Sciences, Development Center of Plant Germplasm Resources, College of Life Sciences, Shanghai Normal University, Shanghai, China; ^2^ Institute of Plant Physiology and Ecology, Shanghai Institutes for Biological Sciences, Chinese Academy of Sciences, Shanghai, China

**Keywords:** Arabidopsis, 5’ splice site selection, cryptic splice site, U1 snRNP, RBP45d, PRP39a, U-rich sequence

## Abstract

Cryptic splice sites in eukaryotic genome are generally dormant unless activated by mutation of authentic splice sites or related splicing factors. How cryptic splice sites are used remains unclear in plants. Here, we identified two cryptic splicing regulators, RBP45d and PRP39a that are homologs of yeast U1 auxiliary protein Nam8 and Prp39, respectively, *via* genetic screening for suppressors of the virescent *sot5* mutant, which results from a point mutation at the 5’ splice site (5’ ss) of *SOT5* intron 7. Loss-of-function mutations in *RBP45d* and *PRP39a* significantly increase the level of a cryptically spliced variant that encodes a mutated but functional sot5 protein, rescuing *sot5* to the WT phenotype. We furtherly demonstrated that RBP45d and PRP39a interact with each other and also with the U1C, a core subunit of U1 snRNP. We found that RBP45d directly binds to the uridine (U)-rich RNA sequence downstream the 5’ ss of *SOT5* intron 7. However, other RBP45/47 members do not function redundantly with RBP45d, at least in regulation of cryptic splicing. Taken together, RBP45d promotes U1 snRNP to recognize the specific 5’ ss via binding to intronic U-rich elements in plants.

## Introduction

Removal of introns from precursor mRNA (pre-mRNA), namely RNA splicing, plays an important role in regulation of gene expression at the posttranscriptional level in eukaryotes. It has been estimated that about 95% of human genes with multiple exons undergo alternative splicing and produce more than one mRNA variants, which significantly expands the human proteome (Chen and Manley, 2009). If pre-mRNA splicing is not accurately processed, it will lead to generation of various altered splicing events and subsequent nonfunctional proteins. In human genetic diseases, 10% are caused by abnormal splicing (Montes et al., 2019). Likewise, regulation of alternative splicing is also one of the important mechanisms regulating plant growth and development in responses to various environmental and developmental cues (Reddy et al., 2013; Staiger and Brown, 2013).

Pre-mRNA splicing is catalyzed by a macromolecular complex called spliceosome, which consists of five core small nuclear ribonucleoprotein particles (U snRNPs) and a number of auxiliary proteins (Chen and Manley, 2009). Coordination of U snRNPs with their auxiliary proteins and many non-snRNP proteins plays an important role in the recognition of splice sites and splicing efficiency. The intron usually contains a number of essential degenerate consensus motifs interacting with U snRNPs, such as at the 5’ and 3’ splice sites (ss), where the most conserved dinucleotides are GU and AG, respectively, and at the branch point site (BP), where A is present 20 to 50 nucleotides (nt) upstream of the 3’ss. In addition, exons and introns may contain *cis*-elements that can enhance or silence splicing and regulate constitutive splicing or alternative splicing *via* interaction with splicing regulatory factors (Chen and Manley, 2009; Lee and Rio, 2015). Interestingly, plant introns have evolved a number of distinct features from those in yeast and animals (Brown et al., 1996). For example, in plants, an intron is usually smaller in size and richer in A and U, the branch point sequence is not obvious, and the pyrimidine tract located between the BPS and 3’ ss is mostly U (Brown et al., 1996). It has been demonstrated that the intronic AU-richness is essential for efficient splicing and 5’ ss selection in dicots (McCullough et al., 1993; Gniadkowski et al., 1996).

RNA splicing is initiated by base-pairing of the 5’ end of the U1 snRNA to the 5’ ss. In yeast and mammals, the 5’ ss consensus sequence for the major U2-type (GT-AG) introns can complement perfectly to that of the U1 snRNA (Roca et al., 2008), which provides a mechanistic explanation for the important role of U1 snRNP in selection of 5’ ss. Besides U1 snRNA, U1 snRNP has seven core subunits called Sm proteins common in all the U snRNPs and three U1-specific proteins (U1-70K, U1A, and U1C) (Will and Luhrmann, 2011). It has been implicated that U1C is essential for U1 snRNP function by interacting with the Sm protein and recognizing the sequence of the 5’ ss (Nelissen et al., 1994; Du and Rosbash, 2002). In contrast, yeast *Saccharomyces cerevisiae* U1 snRNA (568 nts) is significantly larger than mammalian U1 snRNA (De Bortoli et al., 2019), and its U1 snRNP contains seven additional U1 auxiliary proteins including Prp39, Prp40, Snu71, Snu56, Luc7, Prp42, and Nam8 (Li et al., 2017). Among these U1 auxiliary proteins, the yeast Nam8 was reported to directly bind to the uridine (U)-rich sequence downstream of the 5’ ss and U1 snRNP at the same time, so as to effectively help splicing of introns with non-classical 5’ ss sequences (Puig et al., 1999). The human homologous protein of Nam8, TIA-1 (T-cell intracellular antigen-1) has been implicated in U1 snRNP recognition of the 5’ ss and alternative splicing regulation of various pre-mRNA *via* binding to U-rich motifs located downstream of 5’ ss and interaction with the U1C (Le Guiner et al., 2001; Forch et al., 2002). These data indicate that selection of 5’ ss is finely regulated not only by the complementary degree between the 5’ ends of the U1 snRNA and introns but also by many other factors, such as intronic U-rich elements and auxiliary splicing factors. Interestingly, there are eight Nam8 homologs, called the RNA binding protein (RBP) 45/47 family in *Arabidopsis* (Lorkovic et al., 2000; Peal et al., 2011). Recently, one member of the RBP45/47 family, RBP45d, was reported to associate with U1 snRNP *via* interacting with PRP39a and regulate alternative splicing (AS) for a specific set of genes (Chang et al., 2022). In addition, the Arabidopsis genome encodes many homologs of the seven yeast U1 auxiliary proteins (Wang and Brendel, 2004; Reddy et al., 2013). Thus, plant U1 snRNP has been suggested to be similar to or more complex than that in yeast. Although increasing genetic evidence supports that these U1 auxiliary proteins play an important role in plant growth, development and stress response (Wang et al., 2007; Zhan et al., 2015; de Francisco Amorim et al., 2018; Hernando et al., 2019), molecular mechanisms by which they regulate U1 snRNP function remain largely unknown.

There are large numbers of splice sites, known as cryptic splice sites in Eukaryotic genomes, which are generally dormant or used only at low levels unless activated by mutations of nearby authentic splice sites (Roca et al., 2003; Kapustin et al., 2011). Notably, about 9% of all mutations reported in the human gene mutation database are splicing mutations (Anna and Monika, 2018). It is important to be able to predict cryptic splice sites that might be activated in genetic disease so that effective therapeutic strategies can be designed. On the other hand, mutations leading to activation of cryptic splice sites in crops have generated new genetic germplasm resources, such as *waxy* rice, low phytic acid soybean, and pale green Chinese cabbage (Isshiki et al., 1998; Yuan et al., 2012; Zhao et al., 2022). It is generally accepted that cryptic splice sites are suppressed by nearby stronger splice sites and that splice site selection can be viewed as a competition between the various potential splice sites in a pre-mRNA (Kapustin et al., 2011). And it is also proposed that cryptic splice sites are regulated by various RNA binding proteins including heterogeneous ribonucleoproteins (hnRNP) and RNA recognition motif (RRM)-containing SR (serine and arginine-rich) proteins, which are also the alternative splicing regulators (Anna and Monika, 2018). Little is known about splicing factors involved in regulating cryptic splicing in plants. In this study, we showed that Arabidopsis RBP45d facilitates U1 snRNP to preferentially select a cryptic splice site that is activated by mutations of the intronic 5’ ss *via* directly binding to U-rich sequences downstream of the 5’ ss. Our results suggest that RBP45d regulates cryptic splice site selection in plants.

## Results

### An intron mutation leads to activation of two cryptic splice sites in *sot5*

**Figure 1:**
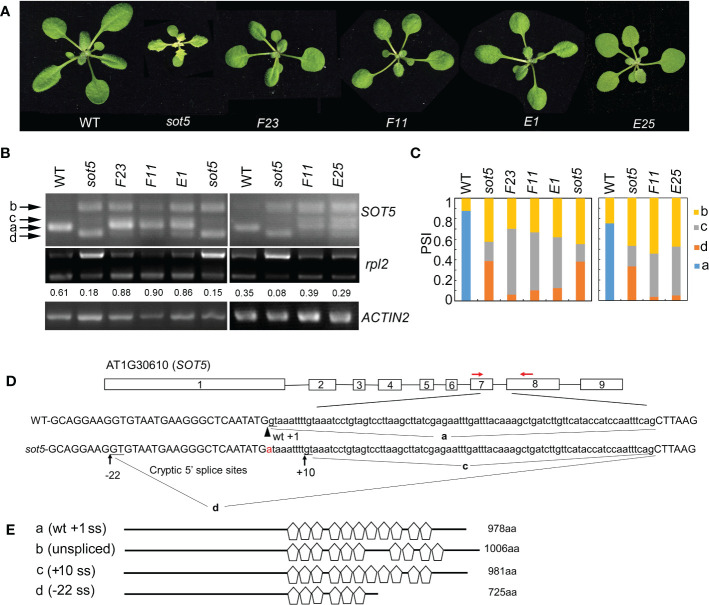
Genetic screening for suppressors of the *sot5* viresent phenotype reveals altered cryptic 5’ splice sites by second mutations. **(A)** Four suppressor lines of *sot5* display WT-like phenotype. The representative 30-d-old plants were shown. **(B)** Semi-quantitative RT-PCR of *SOT5* intron 7 and plastid *rpl2* intron in WT, *sot5*, and the suppressor lines. *ACTIN2* was used as an internal control. Band a shows the WT splicing product. Band b, c, and d correspond to the splicing variants containing intron 7, derived from splice site +10 ss and -22 ss, respectively. The number under the *rpl2* gel band represent the percentage of mature *rpl2* transcripts which quantified by Image J software. **(C)** Quantification of the four bands (a–d) of *SOT5* intron7 RT-PCR products in each genotype showed in **(B)** by Image J software. PSI (percent spliced in index) indicates the percentage of each variant in total transcripts. Three biological replicates (independent pools of aerial parts of plants) were analyzed, and one representative result is shown. **(D)** The schematic diagram of *SOT5* gene structure and intron 7 splicing variants. The black boxes indicate exons and the black lines indicate introns. The authentic 5’ ss in WT is shown by the arrowhead. The G to A point mutation in *sot5* is shown by the red letter. Black arrows indicate two cryptic splice sites, +10 ss and -22 ss. a, c and d indicated the splicing variants corresponding to the bands in **(B)**. Red arrows indicate the primers used for RT-PCR. **(E)** Schematic diagrams of the mutated SOT5 proteins encoded by the variants corresponding to the bands in **(B)**. Pentagons indicate PPR motifs. The numbers indicate the residue number of each protein.

We previously reported that the *Arabidopsis* PPR protein SOT5 (At1g30610), also named EMB2279, is required for intron splicing of the two chloroplast housekeeping genes, *rpl2* and *trnk* (Huang et al., 2018). The *sot5* (*emb2279-3*) mutant contains a G to A mutation at the first base (+1) of the seventh intron and displays a leaf virescent phenotype (**Figures 1A, C**). This point mutation leads to accumulation of the three alternative splicing variants in *sot5*, compared to the wild type (WT) (**Figure 1B**). DNA sequencing of these cloned PCR products indicated that the longest one retained the whole seventh intron with 84 nts (**Figure 1B** and **Figure S1**), the middle transcript retained 9 nts more at the 3’ ss of exon 6 (**Figure 1B** and **Figure S1**), and the shortest one lacked 22 nts at the 3’ end of exon 6 (**Figure 1B** and **Figure S1**). These results indicate that the mutation of the first invariant G at the 5’ ss of intron 7 results in intron retention and activation of two cryptic splice sites named -22 ss and +10 ss (**Figure 1C**). Since the level of band d was much higher than that of band c, the cryptic splicing mainly occurred at -22 ss in *sot5* (**Figure 1B**). Polypeptide prediction indicated that the -22 ss product encodes a truncated protein with only 6 PPR motifs that should be loss of function (**Figures 1D**, **E**); the intron-retained transcript encodes a probably partially functional protein with 10 PPR motifs (Chen et al., 2020); the +10 ss product encodes almost the same protein as the WT one except the seventh PPR motif is slightly changed (**Figures 1D**, **E**, **Figure S2**). Thus, these results interpret well why *sot5* displays a virescent phenotype, but the *emb2279-1* knockout mutant is embryo lethal.

### Genetic screening for suppressors of *sot5* reveals altered cryptic splice sites usage by second mutations

The weak allele *sot5*, in which an intron mutation leads to generation of three abnormal transcripts, provides a good material to study regulation of the cryptic splice site usage. To dissect the genetic pathway for the cryptic splice site selection of *SOT5* intron 7, we screened for *sot5* suppressors which exhibit a WT-like phenotype using the ethyl methanesulfonate (EMS)-mutagenized *sot5* seeds. We obtained a number of WT-like suppressor lines for the *sot5* mutant (**Figure S3**). In this study, four suppressor lines, namely *F23*, *F11*, *E1* and *E25*, were selected for further analysis based on their similar phenotype and alternative splicing pattern of *SOT5* intron7 (**Figures 1A–C** and **Figure S3**). Compared to *sot5*, all these suppressor lines produced a lower level of -22 ss transcripts (band d in **Figure 1B**), but a higher level of +10 ss transcripts (band c in **Figure 1B**), suggesting a positive correlation between the green leaf and the level of the +10 ss transcripts. In *sot5* mutant, the splicing efficiency of plastid *rpl2* and *trnK* intron is significantly decreased (Huang et al., 2018). Indeed, the suppressors of *sot5* were able to recover the splicing defect of plastid *rpl2* intron, which is consistent with their WT-like phenotype (**Figure 1B**). In addition, our genetic analyses showed that the suppressor phenotype was caused by a single gene mutation, and interestingly *F23, F11* and *E1* were allelic while *E25* was mutated in another gene. Taken together, our data suggest that the *sot5* phenotype is suppressed by second mutations that can modulate cryptic splice site selection and splicing efficiency.

### Loss-of-function mutations in *RBP45d* suppress the *sot5* phenotype

**Figure 2:**
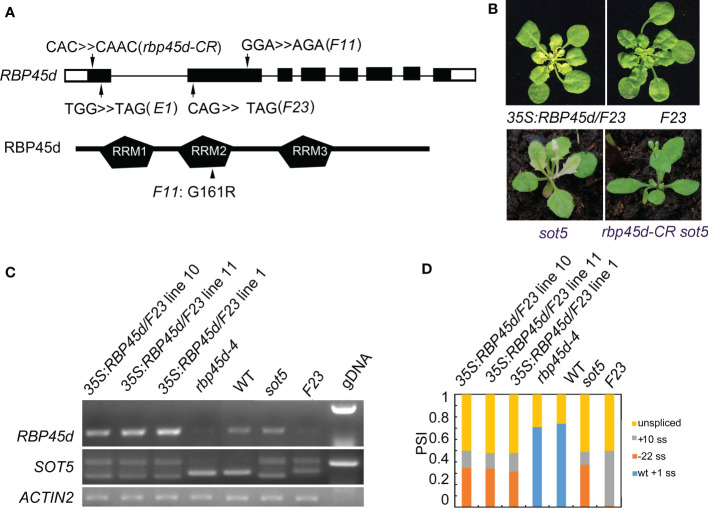
Mutations in RBP45d suppress the *sot5* phenotype. **(A)** Schematic diagrams of *RBP45d* gene structure and RBP45d protein with the three RRM domains. Arrows indicate the mutation sites in *E1*, *F23* and *F11* lines. The upper case letters indicate the mutated genetic codon(s) in *E1* and *F23* which become the stop codons, and that in *F11* which leads to replacement of the amino acid residue from G to R in the conservative region of the RRM2 domain (arrowhead). The black boxes indicate exons in UTR and the black rectangles indicate exons in CDS and the black lines indicate introns. **(B)** Overexpression of *RBP45d* CDS in *F23* restores the phenotype. And the *rbp45d-CR sot5* double mutant showed the WT-like phenotype. One of representative *35S:RBP45d/F23* line was shown. **(C)** RT-PCR analysis of *RBP45d* expression and the splicing products of *SOT5* intron 7 in three *35S:RBP45d/F23* transgenic lines, *rbp45d-4*, WT, *sot5*, and *F23* plants. *ACTIN2* was used as an internal control and gDNA is used as a control. Two biological replicates (independent pools of aerial parts of plants) were analyzed, and one representative result is shown. **(D)** Quantification of the splicing variants from *SOT5* intron 7 shown in **(C)**. PSI (percent spliced in index) indicates the percentage of each variant in total transcripts.

To isolate the suppressor gene in *F23*, we produced a mapping population from the cross between *F23* and Landsberg *erecta* (L*er*). Through Mapping-By-Sequencing (MBS) technique, the gene responsible for phenotypic suppression of *sot5* was mapped to the region with the length ~8 M bp on chromosome 5, which contains 7 point mutations (**Figures S4A, B**). Among these 7 mutations, one stop codon-gain mutation from C to T occurred at 187 bp of the CDS in the locus At5g19350, which encodes RBP45d, a member of the RBP45/47 family with triple RNA recognition motifs (RRM) (**Figures 2A** and **S4B**). Considering that *sot5*-activated cryptic splicing efficiency is altered in *F23*, we speculated that *RBP45d* was the most likely gene to suppress *sot5*. Thus, we sequenced the *RBP45d* gene in *F11* and *E1*, which were genetically testified as *F23* alleles. Indeed, *F11* has a point mutation from G to A at the 481 bp position from the start codon of the *RBP45d* CDS, resulting in the substitution of glycine (G) by arginine (R) at the 161 amino acid residue in the conserved RRM2 domain, whereas *E1* contains a premature stop codon mutation from G to A at the 80 bp position from the start codon of the *RBP45d* CDS (**Figure 2A**).

To verify the mapping result, we overexpressed the native *RBP45d* CDS in *F23*. Our data showed that transgenic lines *35S:RBP45d*/*F23* not only displayed the *sot5* phenotype (**Figure 2B**), but also had the same alternative splicing pattern as the *sot5* mutant (**Figures 2C, D**). In addition, we generated a knockout allele of *RBP45d* (*rbp45d-CR*, with one base insertion at the first exon) in *sot5* mutant background by CRISPR/CAS9 technique, which presented WT-like phenotype (**Figures 2A, B**). These results indicated that the phenotypic recovery of *sot5* is attributed to the loss-of-function mutation in *RBP45d*. We then isolated the single mutant, named *rbp45d-4*, from the F_2_ population of the cross between *F23* and Col-0 and *rbp45d-5* from the F_2_ population of the cross between *F11* and Col-0. RT-PCR analysis showed that *RBP45d* transcripts were almost undetectable in *rbp45d-4* and *F23* (**Figure 2C**), indicating that the point mutation in *rbp45d-4* triggers the nonsense-mediated mRNA decay (NMD) pathway. Taken together, our results imply that *RBP45d* mutations are responsible for the recovery of *sot5* leaf virescence.

### Mutations of other *RBP45/47* members cannot suppress the phenotype of *sot5*

**Figure 3:**
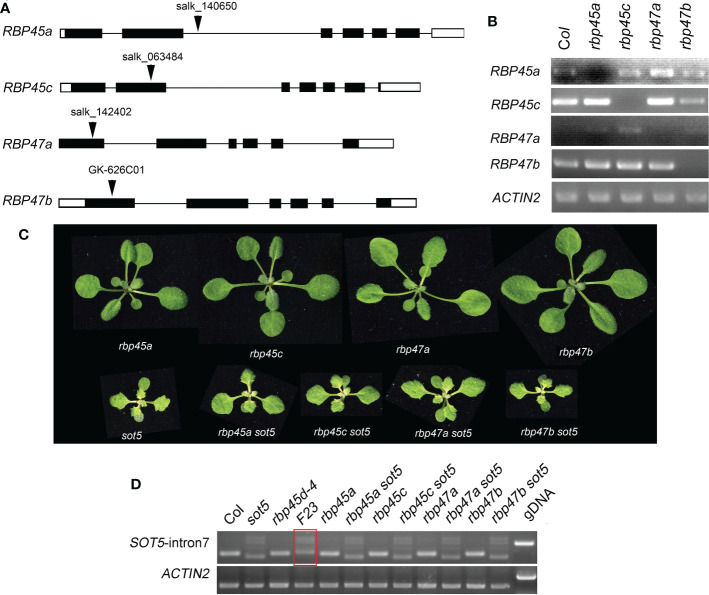
Mutations in other *RBP45/47* paralogs cannot suppress the viresent phenotype of *sot5.* **(A)** Identification of T-DNA insertions in *RBP45a, RBP45c, RBP47a* and *RBP47b* genes. The black boxes indicate exons in UTR and the black rectangles indicate exons in CDS and the black lines indicate introns. Arrowheads indicate the insertional position of T-DNA for each gene. **(B)** RT-PCR analysis of gene expression in the T-DNA knockout mutants. *ACTIN2* was used as an internal control. Two biological replicates (independent pools of aerial parts of plants) were analyzed, and one representative result is shown. **(C)** Phenotypes of single and double mutants as indicated. **(D)** Semi-quantitative RT-PCR showed the splicing pattern of *SOT5* intron 7 in *sot5 RBP45a, sot5 RBP45c, sot5 RBP47a* and *sot5 RBP47b* double mutant when compared with that in *F23* (*sot5 rbp45d-4*). The red box highlighted the altered splicing pattern of *SOT5* intron 7 in *F23.*.

It has been reported that the *Arabidopsis* genome has eight members in the RBP45/47 family (Wang and Brendel, 2004). RT-PCR analysis and eFP Browser (http://bar.utoronto.ca/efp_arabidopsis/cgi-bin/efpWeb.cgi) showed that they are ubiquitously expressed in all organs including roots, inflorescences and leaves (**Figure S5**). Subcellular localization experiment showed that RBP45a, RBP45b, RBP45c, RBP45d and RBP47b were localized in the both nucleus and cytoplasm, while RBP47c’ mainly localized in the cytoplasm (**Figure S6**). The mutant phenotype and physiological function of RBP45/47 family members were not well characterized yet. To know whether other members of the RBP45/47 family can function in the same manner as RBP45d to suppress the *sot5* phenotype, we identified four T-DNA insertion mutants *rbp45a, rbp45c, rbp47a* and *rbp47b* from the ABRC stock (**Figures 3A, B**), and made their double mutants with *sot5* by genetic crossing. All the four single mutants displayed the WT-like phenotype under normal growth conditions, while the double mutants with *sot5* showed the *sot5*-like phenotype (**Figure 3C**). Furtherly, RT-PCR analysis showed that the *SOT5* intron7 splicing pattern was not altered in these mutants when compared with that in *sot5* and *F23* (**Figure 3D**). Therefore, our data indicate that *rbp45a, rbp45c, rbp47a* and *rbp47b* cannot suppress the phenotype of *sot5*.

### RBP45d promote flowering and root growth that different from other *RBP45/47* members

**Figure 4:**
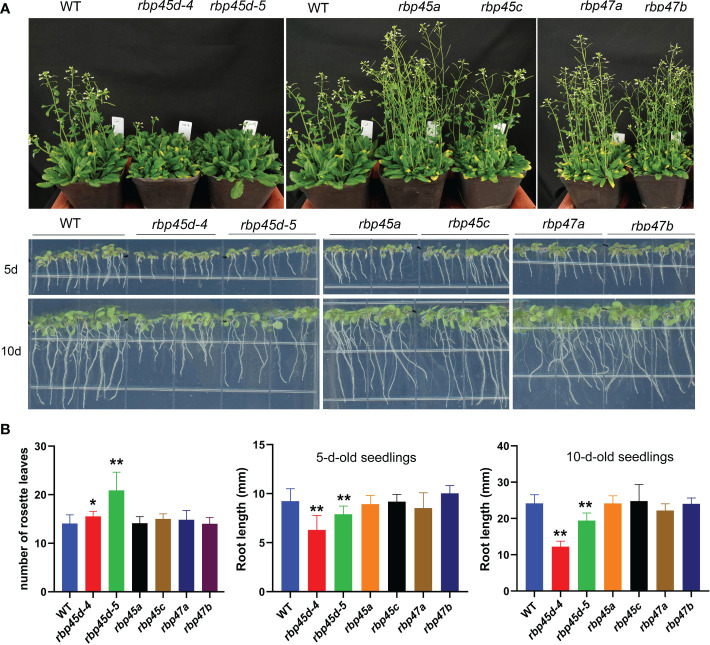
RBP45d promote flowering and root growth that different from other *RBP45/47* members. **(A)** Phenotypic comparison among *rbp45/47* mutants. The upper panel shows the phenotype of 45d-old *rbp45/47* mutants grown under LD conditions (16h light/8h dark photoperiod). The lower panel shows 5-d- and 10-d-old *rbp45/47* mutants vertically grown on 1/2 MS plates. **(B)** The left panel shows the rosette leaf number of each genotype at bolting in LD conditions. The middle and right panels show root length of 5-d and 10-d-old seedlings, respectively. The data are shown means ± SD (n = 20). Student’s *t*-test, * *P* < 0.05, ** *P* < 0.01, compared to WT. The experiments were performed at least three times.

Recently, Chang et al. (2022) and Wang et al. (2021) reported that *RBP45d* regulates temperature-responsive flowering in *Arabidopsis*. In this study, we also observed the later flowering phenotype of *rbp45d* mutants including *rbp45d-4, rbp45d-5* and *rbp45d-CR* (a knockout *rbp45d* mutant created by CRISPR/CAS9 technique) under 16h light/8h dark growth conditions (**Figure 4A** upper panel and **Figure S7**). The number of rosette leaves for *rbp45d* mutants to flower were significantly bigger than that for WT (**Figure 4B** the left panel and **Figures S7B** and **S7C**). However, there were no significant difference in flowering time between WT and other *rbp45/47* plants (**Figures 4A, B**). In addition, we found that loss-of-function mutations in *RBP45d* but not in other *RBP45/47* genes led to the significant shorter primary root phenotype, compared with that of WT, when seedlings were vertically grown on 1/2 MS plates (**Figure 4A** lower panel, **Figure S7**). For 5-day-old seedlings, primary root length of *rbp45d-4* and *rbp45d-5* was 64% and 80% of WT, respectively (**Figure 4A** lower panel and **Figure 4B**). Such a difference in primary root length became much larger between 10-day-old *rbp45d* and WT or other *rbp45/47* seedlings. These results suggest that the biological function of RBP45d is different from that of other RBP45/47 paralogs.

### RBP45d physically interacts with the U1 snRNP core subunit U1C

**Figure 5:**
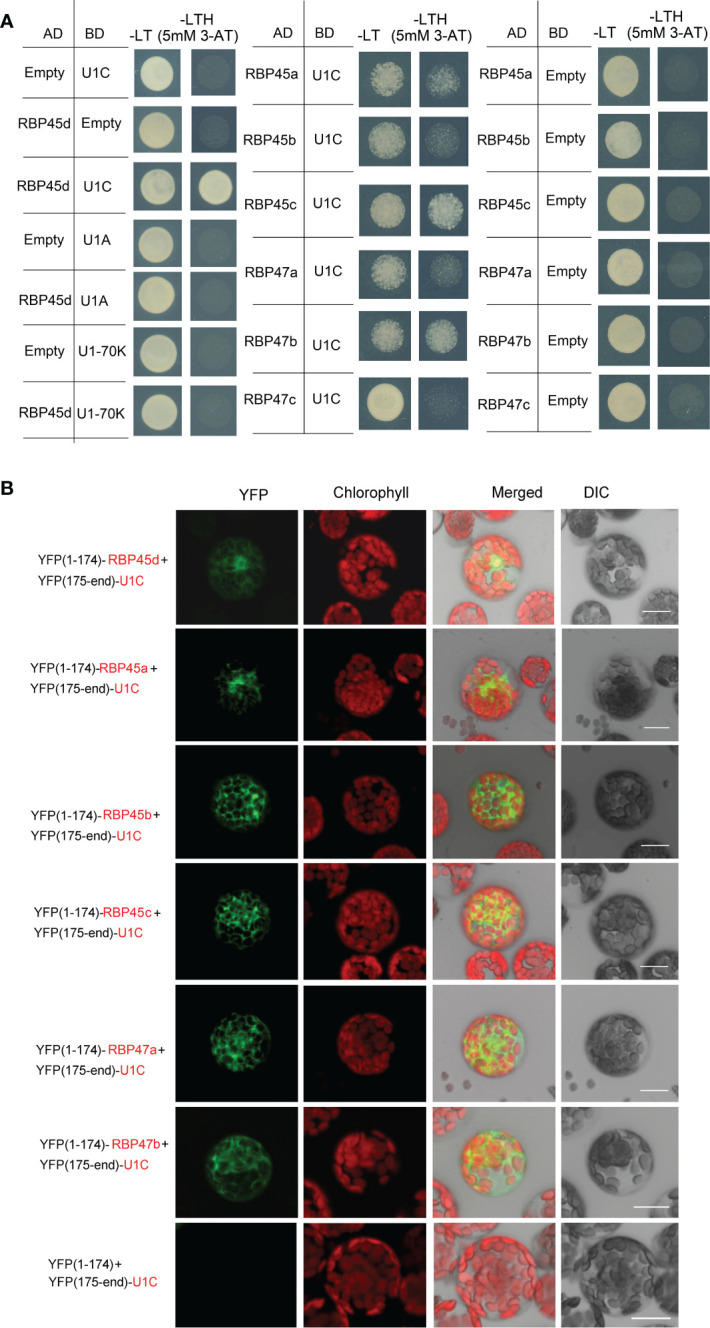
RBP45d and other RBP45/47 members interact with U1C. **(A)** Yeast two-hybrid assays show the interaction between RBP45d and U1C, U1A or U1 70K and the interaction between U1C and RBP45a, RBP45b, RBP45c, RBP47a, RBP47b or RBP47c. The transformed yeast cells were grown on -LT (SD-Leu/-Trp) mediums and -LTH+3-AT (SD- Leu/-Trp/-His with 5 mM 3-AT) mediums. **(B)** BiFC assay of the interaction between U1C and RBP45d, RBP45a, RBP45b, RBP45c, RBP47a or RBP47b. YFP, fluorescence of yellow fluorescent protein; DIC, Differential interference contrast microscopy; Merge, merge of YFP, DIC and chlorophyll. Bar = 15 μm.

RBP45/47 proteins are homologous with yeast Nam8p protein and human TIA-1 protein. It was reported that TIA-1 can directly interact with U1C, the core protein of U1 snRNP (Forch et al., 2002). And the cryo-electron microscopy structure of the yeast *Saccharomyces cerevisiae* prespliceosome revealed that yeast Nam8 protein physically interacts with the U1C. Thus, we proposed that RBP45d may function in a similar manner with TIA-1 and Nam8. We then used yeast two hybrid (Y2H) and BiFC techniques to detect whether RBP45d interacts directly with U1C, U1 70K and U1A of U1 snRNP in *Arabidopsis*. Y2H results showed that RBP45d directly interacted with U1C, but not with U1A and U1 70K (**Figure 5A**). Consistently, BiFC analysis also revealed that RBP45d was bound to U1C in both the nucleus and cytoplasm in *Arabidopsis* protoplasts (**Figure 5B**), which is consistent with their subcellular localization (**Figure S6**). Taken together, these results indicate that RBP45d interacts with U1C *in vitro* and *in vivo*.

Meanwhile, Y2H analysis showed that except for RBP47C, the RBP45/47 members interacted with U1C (**Figure 5A**), whereas BiFC assays indicated that all the tested RBP45/47 paralogs interacted with U1C mainly in the cytoplasm (**Figure 5**). Thus, our results indicate that the biological significance of U1C interaction with RBP45d may differs from that with other RBP45/47 members.

### RBP45d is an intronic U-rich element binding factor

**Figure 6:**
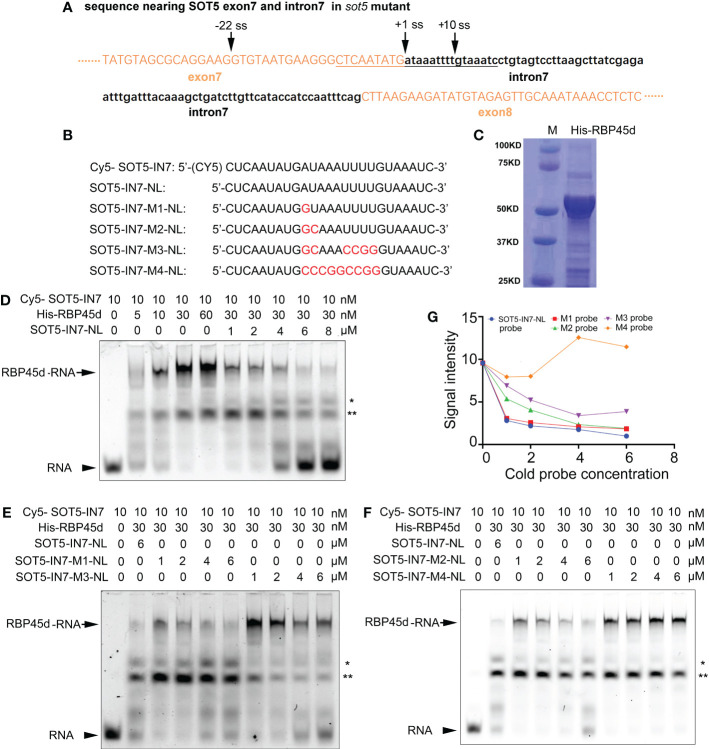
REMSA shows direct binding of RBP45d to the U-rich sequence downstream of the -22 ss of *SOT5* intron 7. **(A)** DNA sequence nearby the cryptic 5’ splice sites in *sot5*. Upper and lower case letters indicate exonic and intronic sequences in *SOT5* gene, respectively. The underlined sequence is used as a template for RNA probe synthesis. Arrows, splice sites. **(B)** Probe names and sequences used for REMSA. The mutation sites in the probes were highlighted with red color. **(C)** Coomassie brilliant-blue staining of the purified His-RBP45d protein separated by SDS-PAGE. **(D)** RBP45d protein can directly bind to the Cy5-SOT5-IN7 RNA probe. **(E)** Competitive binding assay of unlabeled probes SOT5-IN7-M1-NL and SOT5-IN7-M3-NL with the labeled Cy5-SOT5-IN7 probe. **(F)** Competitive binding assay of unlabeled probes SOT5-IN7-M2-NL and SOT5-IN7-M4-NL with the labeled Cy5-SOT5-IN7 probe. Arrows in **(D–F)** show the protein-RNA complex. Arrowheads in **(D–F)** show the free RNA probe. * and ** in **(D–F)** indicate non-specific bands. **(G)** Relative signal intensity of the protein-RNA complex in **(D–F)** was quantified by ImageJ software. The signal intensity of the protein-RNA band in the lane using 6 μM unlabeled SOT5-IN7-NL to compete with the labeled Cy5-SOT5-IN7 probe is artificially set to 1 in **(D–F)**. For REMSA assays, three technical replicates were analyzed, and the results have the same trend. So one representative result is shown.

The above results suggest that RBP45d is recruited to the U1 snRNP *via* interacting with U1C, which is consistent with the working model of yeast Nam8 and human TIA1. It indicates the molecular mechanism of RBP45d regulating 5’ss selection is also conserved in plants. Then we asked whether RBP45d can interact with U-rich sequences. In the *sot5* mutant, the G to A mutation at the 5’ ss of intron 7 results in activation of the two cryptic splice sites (-22 ss and +10 ss) and preferential utilization of the -22 ss (**Figures 1B, C**). In contrast, the absence of RBP45d in the suppressor lines *F23*, *F11* and *E1* leads to preferentially utilize +10 ss (**Figure 1B**), implying that RBP45d plays an important role in controlling the the cryptic splice sites usage. Search for U-rich elements within *sot5* intron 7 indeed revealed the presence of the AUAAAUUUU sequence downstream of the -22 ss (**Figure 6A**). We proposed that RBP45d can bind to this U-rich sequence and subsequently promote the upstream -22 ss selection.

To test this hypothesis, we carried out RNA electrophoretic mobility shift assay (REMSA) with a series of synthesized 26 nt RNA oligo probes including the U-rich element (SOT5-IN7) and the mutated variants (U-poor or U-less, SOT5-IN7-M1~4), and purified His-tagged RBP45d protein from *E. coli* (**Figures 6B, C**). REMSA results showed that RBP45d bound to the Cy5-SOT5-IN7 probe in a dose-dependent manner, and the unlabeled SOT5-IN7-NL probe was able to well compete with the labeled probe in RBP45d binding (**Figure 6D**). Further analysis showed the unlabeled SOT5-IN7-M1-NL and SOT-IN7-M2-NL probes, in which the first A and AU of intron 7 were mutated into G and GC, respectively, still efficiently blocked RBP45d binding to the labeled Cy5-SOT5-IN7 probe (**Figures 6E, F**), whereas the unlabeled SOT5-IN7-M3-NL probe with mutations at the first AU and 4 continuous U of intron 7 significantly reduced the binding affinity to RBP45d (**Figure 6E**). Meanwhile, the unlabeled SOT5-IN7-M4-NL probe completely lost the binding affinity to RBP45d when all the AU-rich element was mutated into the CG-rich one (**Figure 6F**). Quantification of the signal intensity of these protein-RNA bands revealed that the affinity of RBP45d binding to RNA increased with the increase in U and/or A levels of the probes (**Figure 6G**). Thus, our results suggest that RBP45d can bind to the U-rich sequence element, and binding affinity between RBP45d and RNA is dependent on the content of U and/or A.

### RBP45d also modulates cryptic splice site selection in the *clpR4-3* mutant

**Figure 7:**
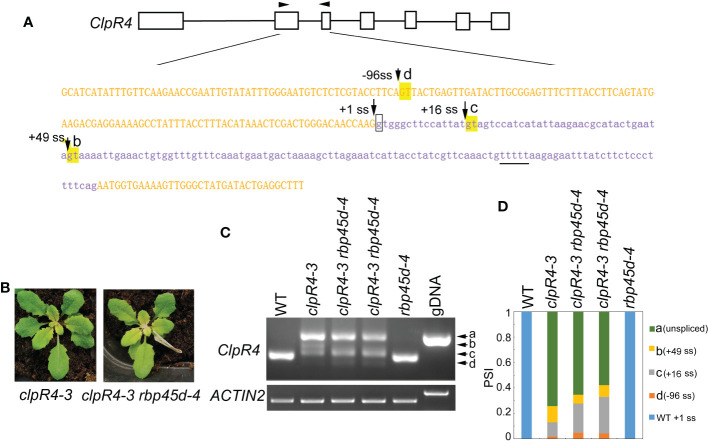
RBP45d regulates the cryptic 5’ splice site selection and splicing efficiency of *ClpR4* intron 2 in *clpR4-3*. **(A)** Schematic diagrams of the *ClpR4* gene and sequences of *ClpR4* exon 2 and intron 2. The black boxes indicate exons and the black lines indicate introns. Upper and lower case letters indicate exon and intron sequences, respectively. The arrows labelled with d, c and b indicate the activated cryptic 5’ splice sites at the -96 ss, +16 ss, and +49 ss, respectively. The arrow labelled with +1 ss indicates the authentic splice site, and boxed g indicates the point mutation in *clpR4-3*. The arrowheads indicate the primers used for RT-PCR. The T-rich sequence downstream the splice site +49ss is underlined. **(B)** Phenotype of *clpR4-3* and *clpR4-3 rbp45d-4* mutants. **(C)** Splicing patterns of *ClpR4* intron 2 in WT, *clpR4-3* and *clpR4-3 rbp45d-4* plants. Band a corresponds to the transcript retained intron 2, whereas band b, c and d correspond to the transcripts spliced at +49 ss, +16 ss and -96 ss, respectively. *ACTIN2* is used as an internal control. Two biological replicates (independent pools of aerial parts of plants) were analyzed, and one representative result is shown. **(D)** Relative levels of the PCR product bands in **(C)** quantified by ImageJ software. PSI (percent spliced in index) indicates the percentage of each variant in total transcripts.

Previously, we reported the G to A mutation at the 5’ ss (+1G-to-A) of *ClpR4* intron 2 in the *clpR4-3* mutant, which displays virescent leaves (Wu et al., 2013). Similarly, the mutation of the authentic splice site leads to retention of intron 2 and activation of the two cryptic splice sites (+16 ss and +49 ss) (**Figure 7A**), producing three different transcripts, named band a, b, and c that are not detected in WT (**Figure 7C**). To examine whether RBP45d is involved in regulation of alternative splicing of *ClpR4* intron 2 in *clpR4-3*, we constructed the *rbp45d-4 clpR4-3* double mutant by genetic crossing. Although *rbp45d-4* was unable to suppress the virescent phenotype of *clpR4-3* (**Figure 7B**), the splice sites usage and splicing efficiency of *ClpR4* mRNA were clearly altered in the double mutant, where band b products significantly decreased while band d products appeared and band c products increased slightly, compared with those in the single *clpR4-3* mutant (**Figures 7C, D**). Sequencing of the cloned PCR products showed that band b, c and d products were derived from the cryptic splice sites +49 ss, +16 ss, and -96 ss, respectively (**Figures 7A, C** and **Figure S8**). These results suggest that the absence of RBP45d in *clpR4-3* allows the U1 snRNP complex to preferentially recognize the cryptic splice sites -96 ss and +16 ss, instead of the cryptic splice site +49 ss. We found a five consecutive U (U-rich) element downstream of the cryptic splice site +49 ss (the underlined letters in **Figure 7A**). It is possible that *RBP45d* binds to this U-rich element in *ClpR4* intron 2, and subsequently promotes the cryptic +49 ss selection in *clpR4-3*.

### Mutations in *PRP39a* suppress the *sot5* phenotype

**Figure 8:**
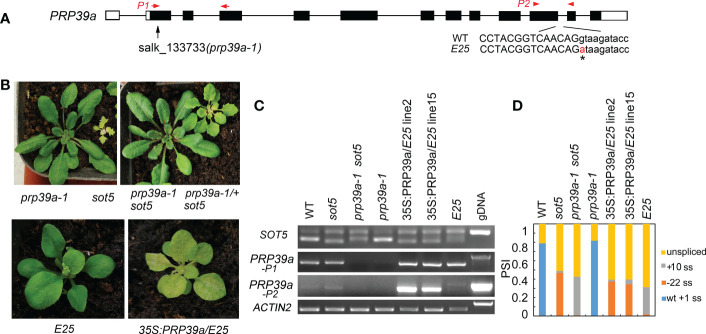
*PRP39a* is a new suppressor gene isolated from *E25.* **(A)** The schematic presentation of the *PRP39a* gene. The black boxes indicate exons and the black lines indicate introns. Black arrow showed the site of T-DNA insertion. The G to A point mutation of *E25* was indicated by *. And red arrow and arrowhead showed the primer pair P1 and P2 used for RT-PCR, respectively. **(B)** Upper panel, phenotypes of *prp39a-1, sot5, sot5 prp39a-1* and *PRP39a-1/^+^ sot5*. Lower panel, phenotypes of *E25* and *35S:PRP39a-gDNA/E25* transgenic plants. **(C)** RT-PCR analysis of the *PRP39a-1, sot5 prp39a-1* and *35S:PRP39a-gDNA/E25* transgenic plants. *ACTIN2* is used as an internal control. Two biological replicates (independent pools of aerial parts of plants) were analyzed, and one representative result is shown. **(D)** Quantification of the *SOT5* PCR product bands in **(C)**. PSI (percent spliced in index) indicates the percentage of each variant in total transcripts.

To clone the suppressor gene in the *E25* line, which displays a similar phenotype to *F23, F11* and *E1* but was not allelic with them, we used the backcrossed F_2_ population between *E25* and *sot5* to clone the suppressor gene *via* the MutMap approach. Our data showed that the suppressor gene was located in a region (~1.3 M) containing 15 candidate genes on Chromosome 1 (**Figure S9**). Interestingly, we found a G to A mutation at the 5’ ss of the eleventh intron in the *PRP39a* gene, which encodes a U1 snRNP auxiliary protein PRP39a (**Figure 8A**). To verify the result, we identified a T-DNA insertion mutant *prp39a* (*prp39a-1*, salk_133733) from the ABRC stock, and made the *sot5 prp39a-1* double mutant by genetic crossing. The double mutant exhibited the same phenotype as *prp39a-1* (**Figure 8B**). Likewise, PCR analysis showed that the pattern of splicing products in *sot5 prp39a* was similar to that in *E25* (**Figures 8C, D**). In addition, we overexpressed the *PRP39a* genomic DNA in *E25*, and found that the phenotype and the splicing pattern of the transgenic lines were the same as those of *sot5* (**Figures 8C, D**). Taken together, our results suggest that *PRP39a* mutations contribute to the suppression of *sot5* in *E25*.

### RBP45d specifically interacts with PRP39a

**Figure 9:**
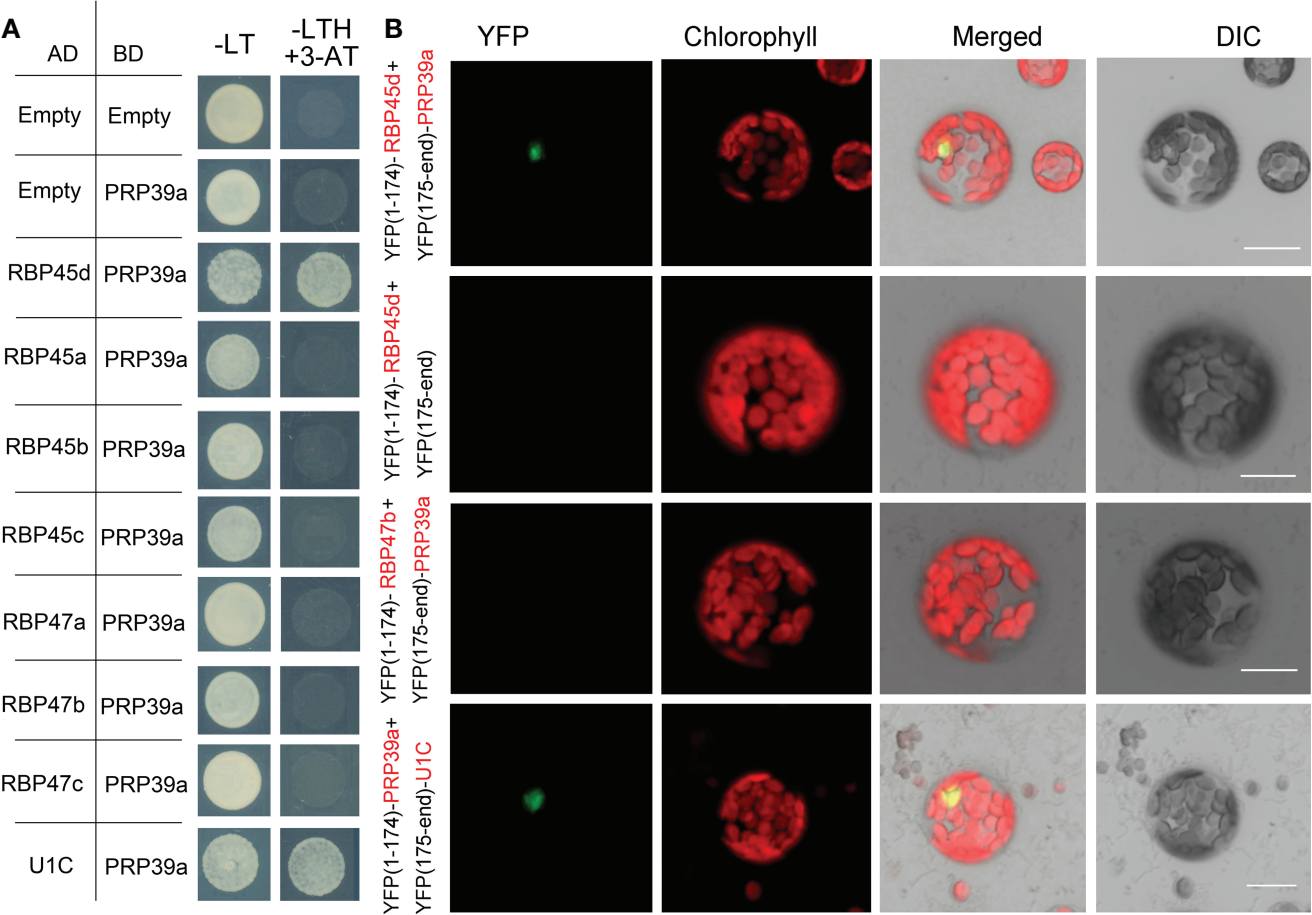
PRP39a interacts with RBP45d and U1C. **(A)** Yeast two-hybrid assays of the interaction between PRP39a and RBP45/47 members or U1C. The transformed yeast cells were grown on -LT (SD-Leu/-Trp) mediums and -LTH+3-AT (SD- Leu/-Trp/-His with 5 mM 3-AT) mediums. **(B)** BiFC assay of the interaction between PRP39a and RBP45d, RBP47b or U1C. YFP, fluorescence of yellow fluorescent protein; DIC, Differential interference contrast microscopy; Merge, merge of YFP, DIC and chlorophyll. Bar = 15 μm.

The yeast Prp39 and it paralog Prp42 form the scaffold connecting auxiliary proteins associated with U1 snRNP to the core (Li et al., 2017). RT-PCR analysis and eFP browser showed that Arabidopsis *PRP39a* is ubiquitously expressed (**Figure S10A**). However, PRP39a protein specifically localized in nucleus, which is different from that of RBP45d (**Figure S10B**). Our genetic screening of *sot5* suppressors indicated that RBP45d and PRP39a act in the same genetic pathway with regard to *SOT5* intron 7 alternative splicing. In addition, the *prp39a-1* mutant exhibited the same shorter primary root phenotype as *rbp45d*, compared with that of WT (**Figure S11**). We then examined whether PRP39a and RBP45d physically interact each other *in vitro* and *in vivo*. Y2H results showed that PRP39a directly interacted with RBP45d, but not with other RBP45/47 members (**Figure 9A**). Consistently, BiFC analysis showed that PRP39a and RBP45d interacted specifically in the nucleus (**Figure 9B**), which is consistent with the nucleus localization of PRP39a (**Figure S10**). However, we did not detect the fluorescence signal between PRP39a and RBP47b (**Figure 9B**), indicating that they are not associated each other *in vivo*. These results are consistent with previously reported that the role of RBP45d in alternative splicing relies on its specific interaction with PRP39a (Chang et al., 2022). Meanwhile both Y2H and BiFC results showed that PRP39a was able to interact with U1C in the nucleus (**Figure 9B**). Taken together, our results suggest that Arabidopsis RBP45d, PRP39a and U1C function in a similar way to that in yeast with regard to alternative splicing regulation.

## Discussion

In this study, we used a powerful genetic approach to screen phenotypic suppressors of the *sot5* mutant, which contains a G to A mutation at the first nucleotide of intron 7 (Huang et al., 2018), and isolated two suppressor genes, *RBP45d* and *PRP39a*. RBP45d and PRP39a are yeast Nam8 and Prp39 homologs, respectively, which are auxiliary components of the U1 snRNP complex. Although they have been demonstrated to play important roles in alternative splicing in yeast, human and Arabidopsis (Puig et al., 1999; Le Guiner et al., 2001; Forch et al., 2002; Chang et al., 2022), their function in cryptic splice site selection was not reported yet. Here we collected several lines of evidence substantially supporting that RBP45d and PRP39a are two *trans*-acting splicing factors that facilitate U1 snRNP to select specific 5’ ss. First, loss-of-function mutations in *RBP45d* and PRP39a significantly alter 5’ cryptic splice site usage in the *sot5* mutant. Second, RBP45d physically interacts with the core subunit U1C of U1 snRNP and the U1 auxiliary factor PRP39a. Third, RBP45d can bind to U-rich sequences and its binding affinity is positively correlated to the U content of the sequence. Taken together, our data suggest that RBP45d promotes 5’ ss selection by directly binding to its downstream U-rich *cis*-element and then recruits U1 snRNP to the splice site by interacting with both PRP39a and U1C.

### The working model of RBP45d and PRP39a in facilitating U1 snRNP to select specific 5’ ss

**Figure 10:**
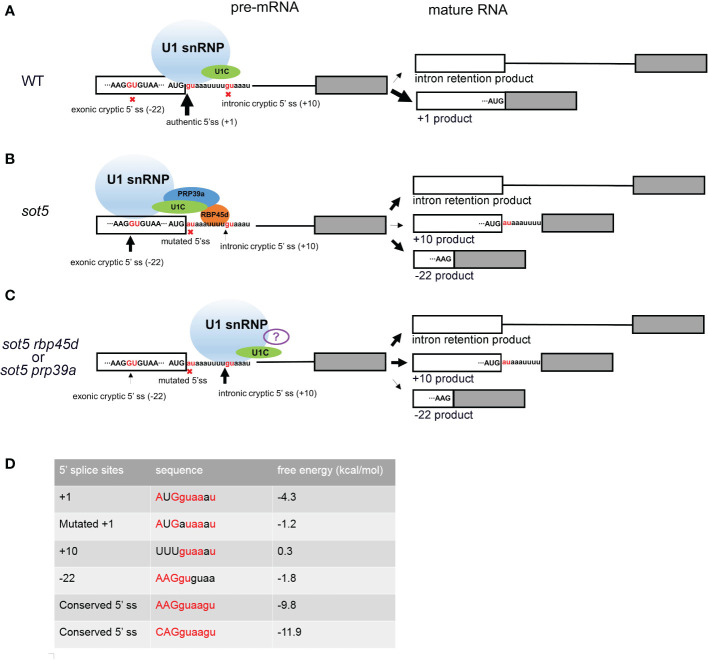
Working model of RBP45d and PRP39a in 5’ cryptic splice site selection. **(A)** In WT plant, most *SOT5* transcripts are excised intron 7 and only a very small proportion of *SOT5* transcripts retained intron 7. Since the authentic 5’ ss is strong enough to well base pair with U1 snRNA and recruit U1 snRNP, no cryptic 5’ ss nearby were used. **(B)** In *sot5*, the mutation of the authentic 5’ ss of *SOT5* intron7 led to the reduced base pairing with U1 snRNA, so that the exonic and intronic cryptic 5’ ss nearby are activated. Beside, nearly half transcripts retained intron 7. In this scenario, RBP45d binds to the U-rich element located downstream of the exonic cryptic 5’ ss and recruits U1 snRNP to select the exonic cryptic 5’ ss by physically interacting with both U1C and PRP39a. It is also likely that the RBP45d bound to the U-rich element which is proximal to the intronic cryptic 5’ ss masks this 5’ ss. **(C)** When RBP45d or PRP39a is absent or disrupted, the masked intronic cryptic 5’ ss is exposed and is preferred by the U1 snRNP complex (lacking PRP39a and RBP45d or with some other unknown splicing factors). The white and gray boxes indicate the exon 7 and exon 8 of *SOT5* transcripts, respectively. The black lines indicate the intron 7. The sequence nearby the 5’ ss of intron 7 was shown and the invariant GU dinucleotides were colored by red. The nine nucleotides AUGguaaau indicate the authentic 5’ ss in WT. The nine nucleotides AUGauaaau indicate the mutated 5’ ss in *sot5*. The nine nucleotides AAGGUGUAA indicate the exonic cryptic 5’ ss in *SOT5* exon 7. The nine nucleotides uuuguaaau indicate the intronic cryptic 5’ ss in *SOT5* intron7. The red crosses indicate the splice sites were not used. The black arrows indicate the usage of the 5’ ss and the thickness of the arrows indicate the frequency of the splice site usage. **(D)** Comparison of the conserved degree of these 5’ ss and the free energy of the RNA duplex formed by the 5’ ss based pairing with the 5’ end of U1 snRNA. The conserved 5’ ss sequence well based paired with the 5’ end of U1 snRNA, the free energy of the RNA duplex is low. On the contrast, if the 5’ ss sequence did not based paired with the 5’ end of U1 snRNA, the free energy of the RNA duplex is relatively high. The conserved nucleotides are highlighted by red color. The free energy was calculated by The RNAeval web server (http://rna.tbi.univie.ac.at//cgi-bin/RNAWebSuite/RNAeval.cgi).

It is well known that U1 snRNP plays a critical role in initiation of 5’ ss recognition by base pairing of U1 snRNA and 5’ ss consensus sequence. And several U1 snRNP auxiliary proteins such as Nam8, Luc7 and RBM25 can also influence 5’ ss selection, especially when the 5’ss sequence are not conserved (Fortes et al., 1999; Puig et al., 1999; Forch et al., 2002; Puig et al., 2007; Zhou et al., 2008). Based on the data reported previously, the Arabidopsis U1 snRNP component RBP45d is required for alternative splicing for a set of genes (Kanno et al., 2020; Chang et al., 2022). Consistently, the two RBP45d homologs, yeast Nam8 and human TIA-1, are implicated in alternative splicing *via* binding to the U-rich region downstream of a weak 5’ ss (Puig et al., 1999; Le Guiner et al., 2001; Aznarez et al., 2008; Qiu et al., 2011). The similar molecular function of Nam8, TIA-1, and RBP45d is attributed to their highly conserved domains including the three RRM domains and the C-terminal Gln-rich tail. Cryo electron microscopy (cryo-EM) analysis of prespliceosome structure revealed that Nam8 or TIA-1 binds to U-rich sequences through the RRM2 domain to define the use of weak 5’ ss, and interacts with the U1C-terminal region near the U1-5’ ss helix *via* the RRM3 domain (Plaschka et al., 2018). Recently, Cryo-EM structure of the yeast U1 snRNP showed that the alternative splicing factors Prp39 and Prp42 form heterodimer that acts as a central scaffold connecting all auxiliary proteins associated with U1 snRNP to the core (Li et al., 2017). In human, there is no Prp42 homolog, but the Prp39 homolog PrpF39 forms a homodimer that acts as the Prp39/Prp42 heterodimer in U1 snRNP. In addition, Nam8/TIA-1 interacts with the Prp39/Prp42 heterodimer by its RRM3 domain and C-terminal tail (Plaschka et al., 2018). The structural and biochemical data provide insight into alternative splicing mediated by Nam8/TIA-1 and Prp39/prp42. Although the U1 snRNP complex and structure remain to be dissected in plants, most of the counterparts of yeast U1 snRNP core subunits and auxiliary proteins are present in Arabidopsis and encoded by multiple genes (Wang and Brendel, 2004). Considering that the basic mechanism of pre-mRNA splicing is highly conserved in all eukaryotic organisms, we propose that RBP45d functions as a U1 snRNP auxiliary factor in the same way as Nam8/TIA-1. As expectedly, our data showed that RBP45d can not only bind to U1C (the core protein of U1 snRNP) and PRP39a (the scaffold protein connecting the auxiliary proteins to the core), but also to the downstream U-rich RNA sequence near 5’ ss of *SOT5* intron 7. Accordingly, we suggest a working model that RBP45d can recruit spliceosome to specific 5’ ss and enhance the stability of spliceosome and pre-mRNA complex by directly binding to U-rich RNA sequences downstream of 5’ ss (**Figure 10**).

### RBP45d and PRP39a regulate cryptic splice site selection in 5’ ss mutants

Cryptic splice sites are repressed by complicated mechanisms in eukaryotes. Study of many model organisms revealed that when the authentic splice site is mutated, the cryptic splice sites close to the authentic splice site will be activated (Roca et al., 2003). Usually, there are multiple cryptic splice sites nearby. The mechanism of which cryptic splice site is preferentially selected is not very clear. It is reported that according to the conservation degree of the sequence near 5’ ss, the strong or weak 5’ ss can be predicted. The more conservative the motif is (i.e. it can be perfectly base paired with U1 snRNA), the stronger the splice site is. And the strong splice site will be preferentially and efficiently spliced. On the contrary, the less conservative the motif, the weaker the splice site is (Roca et al., 2003; Dawes et al., 2022). However, when there are AU-rich or U-rich sequences in the intron, this prediction is not accurate, because the U-rich sequence downstream of the weak splice site will be bound by specific splicing factors, such as Nam8p or TIA-1, and the weak splice site will be activated in this scenario. It is reported the AU-rich sequences in plant introns are more abundant and the sequence of 5’ ss is more variable. Therefore, the selection of plant splice sites may be more dependent on the location and abundance of AU- rich or U-rich sequence of introns (McCullough et al., 1993). But what *trans-acting* factor that acting on the AU- rich or U-rich sequence in plants remains unclear. Our study showed that RBP45d is a such splicing factor for U-rich introns splicing in plants. When the 5’ ss of *SOT5* intron7 is mutated, two cryptic splice sites at position - 22 and + 10 are activated, but the + 10 ss (splice site) is much weaker than the - 22 ss. RBP45d or PRP39a mutation reverse the usage of two cryptic splice sites (**Figure 10**). By carefully analyzing the sequences downstream of these two cryptic splicing sites, we found that there is a U- rich sequence that can be bound by RBP45d downstream of - 22 ss. In addition, we demonstrated that RBP45d physically interacted with PRP39a, consistent with the results that they act on the same genetic pathway.

### The possible different roles of U1 snRNP auxiliary proteins RBP45d, PRP39a, LUC7 paralogs and RBM25 in regulating 5’ ss selection

CryoEM revealed that yeast U1 snRNP contains seven additional U1 auxiliary proteins including Prp39, Prp40, Snu71, Snu56, Luc7, Prp42, and Nam8 (Li et al., 2017). Yeast Luc7, a Zinic-finger RNA binding protein, is also involved in 5 ‘ss recognition (Fortes et al., 1999). It is suggested that interaction of yeast Luc7 with the upstream exon stabilizes the pre-mRNA–U1 snRNP interaction. Similarly, human hLuc7A affects 5’ss selection possibly *via* the formation of a network of protein-RNA interactions that stabilize pre-mRNA–U1 snRNP interaction and thus formation of the commitment complex. (Puig et al., 2007). There are three *LUC7* paralogs (*LUC7A, LUC7B* and *LUC7RL*) in Arabidopsis genome and they specifically promote splicing of a subset of terminal introns and affects plant development and adaptation to stress (de Francisco Amorim et al., 2018). Whether the LUC7 family members acted at the same pathway with RBP45d and PRP39a remains unclear. Arabidopsis RBM25 (homolog of yeast Snu71) was aslo an U1 auxiliary protein, but it acts differently with RBP45d to regulate alternative splicing (Kanno et al., 2017). Human RBM25 was reported to specifically bind to *Bcl-x* RNA through a CGGGCA sequence located within exon 2 and enhance *Bcl-x_s_* 5’ ss selection and thus regulate apoptotic cell death (Zhou et al., 2008). In addition, human RBM25 was found to associate with a U1 snRNP associated factor, hLuc7A by Co-IP (Zhou et al., 2008). These results indicate that the different U1 snRNP auxiliary protein may bind different *cis*-element in exons or introns to stabilize U1 snRNP and pre-mRNA complex and differentially regulate the alternative splicing of the pre-mRNA. The relationship among RBP45d, RBM25, and LUC7 family members remains to be elucidated.

It is noted that in Arabidopsis, although some of the U1 snRNP auxiliary proteins are encoded by two or more genes, they generally do not function redundantly in pre-mRNA splicing. RBP45d is a good example. Besides, Kanno et al., 2017 reported that Arabidopsis *PRP39a* mutation changes the alternative splicing of GFP reporter gene, while *PRP39b* cannot, indicating that they have different functions in splicing regulation. Probably, *LUC7A, LUC7B* and *LUC7RL* may also preserved their unique function in regulating pre-mRNA splicing. How these Arabidopsis U1 snRNP auxiliary proteins regulate 5’ss selection remains to be explored.

### The distinct function of RBP45d from other members

There are eight paralogs in the Arabidopsis RBP45/47 family. Whether they function redundantly or not remains unclear. Here we showed that RBP45d functions differently with other RBP45/47 members. The specificity of RBP45d in pre-mRNA splicing is supported by the following evidence. The *sot5* phenotype can be rescued by mutations in *RBP45d* but not in other *RBP45/47* members. Only RBP45d is interacted with PRP39a, since other RBP45/47 members lack the long C-terminal tail that was demonstrated to play an important role in protein-protein interaction between RBP45d and PRP39a (Chang et al., 2022). Furthermore, we observed that *rbp45d* mutants have short primary root and late flowering phenotypes, which are not present in other *rbp45/47* mutants (**Figure 4** and **Figure S7**). However, we found some common features in all the RBP45/47 members. For example, they are localized in both the nucleus and cytoplasm (**Figure S5**), and they all interact with the U1 snRNP core subunit U1C (**Figure S6**). Thus, it is likely that other RBP45/47 members are also involved in splicing regulation through the mechanisms different from those of RBP45d. It is noted that most of the RBP45/47 members including RBP45d are localized both in nucleus and cytoplasm, indicating that they may also play roles in mRNA regulation in cytoplasm. It will be interesting to investigate biological functions of other RBP45/47 members as well as the molecular mechanisms by which they act in plants.

It is interestingly that the Tabaco NtRBP45 was recently reported to facilitate post-transcriptional gene silencing (PTGS) by promoting siRNA production (Zhang et al., 2020). While the Aarabidopsis RBP45d was reported to promote RNA-directed DNA methylation (RdDM), similar to the known components of RdDM (NRPD2A, RDR2, AGO4 and AGO6). It is suggested that RBP45d facilitates siRNA production by stabilizing either the precursor RNA or the slicer protein (Wang et al., 2021). Several research groups have also found mutations of some mRNA splicing factors reduced the level of siRNA dependent on POL IV, but it is unclear how these splicing factors affect the level of siRNA (Ausin et al., 2012; Dou et al., 2013; Huang et al., 2013; Zhang et al., 2013). It will be interesting to explore how the splicing factors function in RNA-directed DNA methylation in the future.

## Materials and methods

### Plant materials and growth conditions

The Arabidopsis ecotype Columbia-0 (Col-0) was used as the wild type (WT) in this study. The mutants *sot5* and *clpR4-3* were previously described (Huang et al., 2018, Wu et al., 2013). The *prp39a-1* (salk_133733), *rbp45a* (salk_140650)*, rbp45c* (salk_063484)*, rbp47a* (salk_142402), and *rbp47b* (GK-626C01) mutants were obtained from the ABRC stock center (https://abrc.osu.edu/) and were genotyped by PCR using gene specific primers (**Table S1**). Double mutants *prp39a-1 sot5, rbp45a sot5*, *rbp45c sot5 rbp47a sot5 rbp47b sot5*, and *clpR4-3 rbp45d-4* were identified from F_2_ generations derived from crosses between single mutants by the PCR-based genotyping procedure. Seeds were surface-sterilized by 75% ethanol and stratified at 4°C for 3 days, and then sown onto half-strength Murashige and Skoog (MS) medium with 0.5% sucrose. Seeds were also directly sown in soil and grown in a phytotron with long-day conditions (16 h light/8 h dark) and light intensity (100 μmol photons m^-2^ s^-1^) at 22°C.

### EMS mutagenesis, gene cloning, plasmid construction and transformation

In order to obtain *sot5* suppressors, *sot5* seeds were ethyl methanesulfonate (EMS) mutagenized (0.1%). About 30000 M_2_ plants from 5000 M_1_ lines were screened. Several suppressor lines that displayed WT-like green leaves were obtained from the M_2_ generation. To clone *F23*, we produced an F_2_ population from the cross between *F23* and Landsberg *erecta* (L*er*). In the F_2_ population, about eighty *F23* plants were pooled for Whole Genome Resequencing and Mapping-By-Sequencing (MBS) analysis (OE Biotech Co.,Ltd). To clone the *E25* gene, we crossed the *E25* with *sot5* and generated the BC_1_ F_2_ population. About thirty *E25* plants were pooled for Whole Genome Resequencing and Mutmap analysis (Biomarker Technologies).

The *RBP45d* coding DNA sequence (CDS) and *PRP39a* genomic DNA were cloned into the pENTR SD/D-TOPO entry vector (Invitrogen), and then recombined into the pGWB2 destination vector, respectively. The destination vector containing *RBP45d* was transformed into *F23* mutant and the destination vector containing *PRP39a* was transformed into *E25* mutant according to the method as our previously described (Huang et al., 2018). To obtain recombinant RBP45d protein, the *RBP45d* CDS was transferred into the pet 51b vector and then was fused with His tag at its N-terminal.

### Generation of *rbp45d-CR* mutant lines by CRISPR/Cas9-mediated genome editing

The *rbp45d-CR* mutant line was generated by CRISPR/Cas9-mediated genome editing. The sgRNA sequences were designed by the online software CRISPR-P v2.0 (Liu et al., 2017) and CRISPR-GE (Xie et al., 2017) to target exon1 of the *RBP45d* gene. The binary vector pYAO-Cas9-1300 (Feng et al., 2018) was linearized using *Bsa*I (ThermoFisher Scientific, UK) endonuclease. Two complementary sgRNA nucleotides were synthesized and annealed to generate double-strand DNA with appropriate overhangs on both ends and then inserted into the pYAO-Cas9-1300 vector. The vector was transformed into the *Agrobacterium tumefaciens* strain GV3101 competence cells. And the GV3101 with the vector was transformed into the Col-0 plants by the floral dip method. The transgenic plants of T_1_ generation were screened by Hygromycin B resistance, DNA from positive lines was extracted and PCR and sequencing were performed to identify mutations in sgRNA target sequences. Finally, we screened the Cas9-free background and homozygous *RBP45d* gene editing lines in T_2_ generation plants to obtain *rbp45d-CR* mutant lines.

### Analysis of RT-PCR and sequencing of the cloned PCR products

For RT-PCR, total RNAs were extracted from seedlings or different plant tissues by RNA Easy Fast Plant Tissue Kit (TIANGEN, DP452) according to the manufacturer’s instructions. DNase I treatment and RT-PCR analysis were conducted as previously described (Huang et al., 2018). Semi-quantitative RT-PCR was carried out using the gene-specific primers (**Table S1**). To identify the splicing variants, the PCR products amplified by the primers spanning the introns were purified and cloned into pMD19-T vector (TaKaRa, D102A). About 10~20 clones of each PCR products were sequenced and aligned with the corresponding genes by SnapGene software.

### Subcellular localization of RBP45/47 members and PRP39a fused with YFP and BiFC assay

To express YFP-fused protein, the full length CDS of the *RBP45a, RBP45b, RBP45c, RBP45d, RBP47a, RBP47b, RBP47c, RBP47c’, PRP39a* and *U1C* genes were amplified from the cDNA of WT seedlings using the primers (**Table S1**). The sequences were cloned into the pENTR SD/D-TOPO entry vector (Invitrogen) and then recombined into the p2YWG7 vector (http://gateway.psb.ugent.be/vector/show/p2GWY7/search/index/) or BiFC vctors pE3130 and pE3136 (http://www.bio.purdue.edu/people/faculty/gelvin/nsf/protocols_vectors.htm). The purified plasmid was transformed into the *Arabidopsis* protoplasts according to the method (Wu et al., 2009). The BiFC vector combinations are indicated in the **Figures 5** and **10**.

### Yeast two hybrid

The CDS of *RBP45a, RBP45b, RBP45c, RBP45d, RBP47a, RBP47b, RBP47c, PRP39a U1A, U1C* and *U1-70K* were clone into the pENTR vector and then recombined into pDEST22 and pDEST32 destination vectors, respectively. For yeast two-hybrid assays, plasmids were transformed into yeast strain AH109 by the lithium chloride–polyethylene glycol method according to the manufacturer’s manual (Clontech). The transformants were selected on SD-Leu-Trp plates. The protein–protein interactions were tested on SD-Trp-Leu-His plates with or without 5mM 3-amino-1,2,4-triazole (3AT).

### Protein expression, purification and RNA electrophoretic mobility shift assay

The RBP45d CDS was cloned into the expression vector pet51b, and protein expression was induced using 0.5 mM isopropyl-b–D–thiogalactopyranoside at 16°C for 12 h in E. coli strain Rosetta. The protein was purified as described by Zhu et al. (2020). The RNA probes were chemically synthesized, and their 5’-end were labeled by Cy5 (Sangon Biotech (Shanghai) Co., Ltd). The sequences of the probes were as follows:

Cy5-SOT5-IN7: 5’-(CY5) CUCAAUAUGAUAAAUUUUGUAAAUC-3’;

The corresponding non-labeled and mutated probes were also chemically synthesized for competition assays.

SOT5-IN7-NL: 5’-CUCAAUAUGAUAAAUUUUGUAAAUC-3’
SOT5-IN7-M1-NL: 5’-CUCAAUAUGGUAAAUUUUGUAAAUC-3’
SOT5-IN7-M2-NL: 5’-CUCAAUAUGGCAAAUUUUGUAAAUC-3’
SOT5-IN7-M3-NL: 5’-CUCAAUAUGGCAAACCGGGUAAAUC-3’
SOT5-IN7-M4-NL: 5’-CUCAAUAUGCCCGGCCGGGUAAAUC-3’

For EMSA assay, RBP45d protein and RNAs were incubated at room temperature for 30 min in the reaction solution (10 mM HEPES pH 7.3, 20 mM KCl, 1 mM MgCl2, 1 mM dithiothreitol, 5% glycerol (v/v) and 0.1 μg tRNA). The reactants were analyzed using a native polyacrylamide gel, and signals were detected by Azure Biosystems C600.

## Data availability statement

The original contributions presented in the study are included in the article/**Supplementary Material**. Further inquiries can be directed to the corresponding author. The accession numbers of the gene described in the study are listed in **Table S2**.

## Author contributions

WH and JH conceived and designed the research. WH, LZ, YajZ, JC, YawZ, FL and XC performed the experiments. WH and JH supervised the experiments and wrote the article. All authors contributed to the article and approved the submitted version.
