# Supplementary material for: A genetic screen in Arabidopsis reveals the identical roles for RBP45d and PRP39a in 5’ cryptic splice site selection
Source: Front Plant Sci. 2022 Dec 22;13:1086506. doi: 10.3389/fpls.2022.1086506 (PMC9813592; doi:10.3389/fpls.2022.1086506)
Supplement: Supplementary file 1 [file DataSheet_1.pdf]

Supplementary Material

1 Supplementary Figures

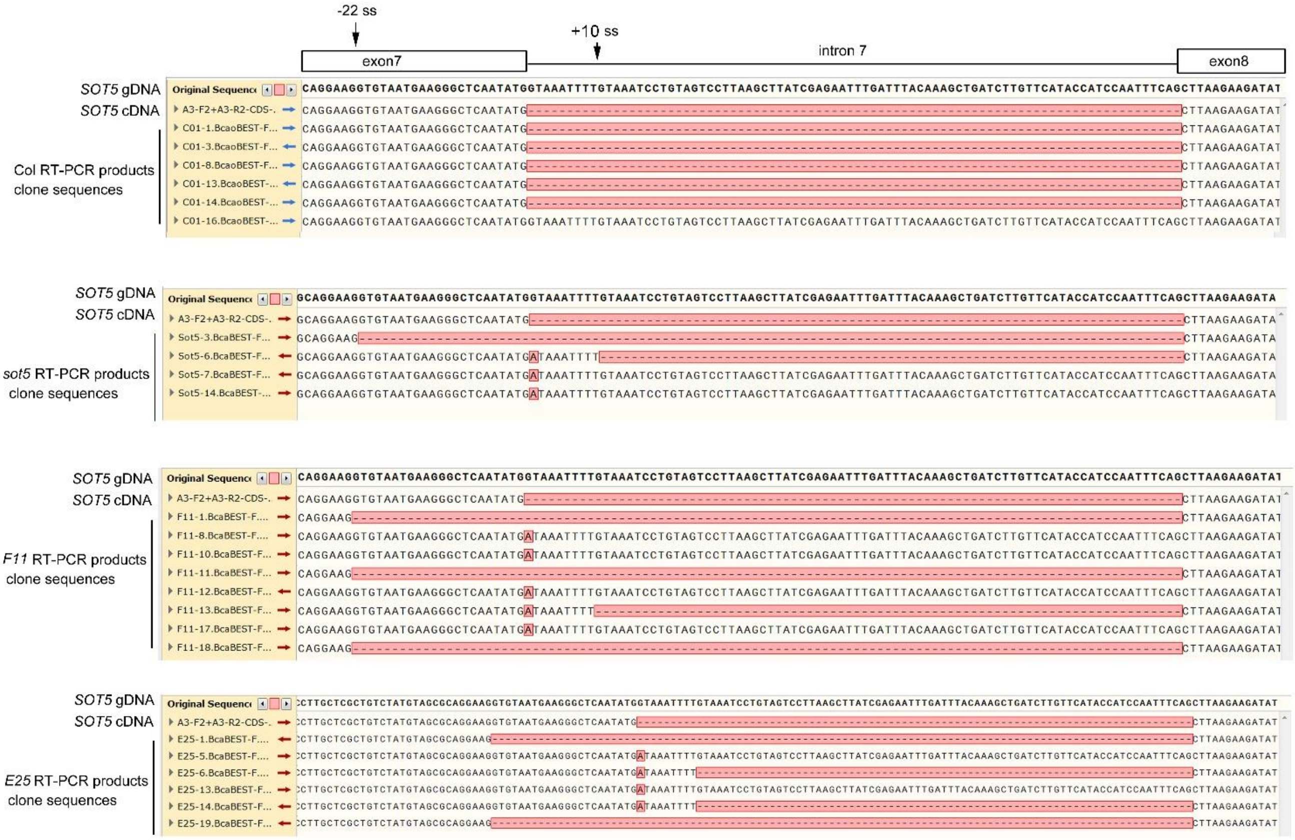

Figure S1. Sequence analysis of the cloned RT-PCR products amplified across *SOT5* intron 7 in WT, *sot5* and the suppressor lines *F11* and *E25*. The DNA sequences from different clones are aligned by SnapGene software. The sequences nearing *SOT5* intron 7 are showed.

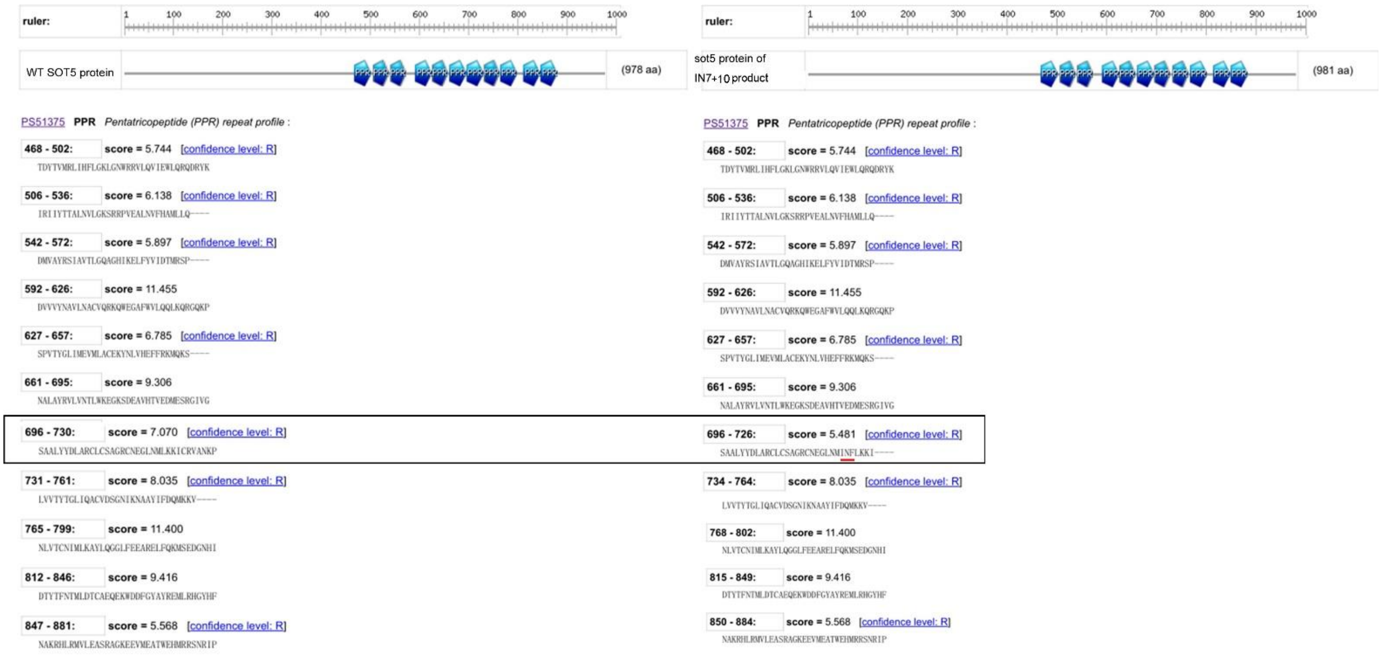

Figure S2. Comparison of the two SOT5 proteins encoded by IN7 wt +1 ss and IN7 +10 ss products. The domains of different protein variants were predicted by Prosite （https://prosite.expasy.org/). The 981 aa sot5 protein is generally as same as the WT one (978 aa) except that the seventh PPR domain is slightly altered (marking by the black box).

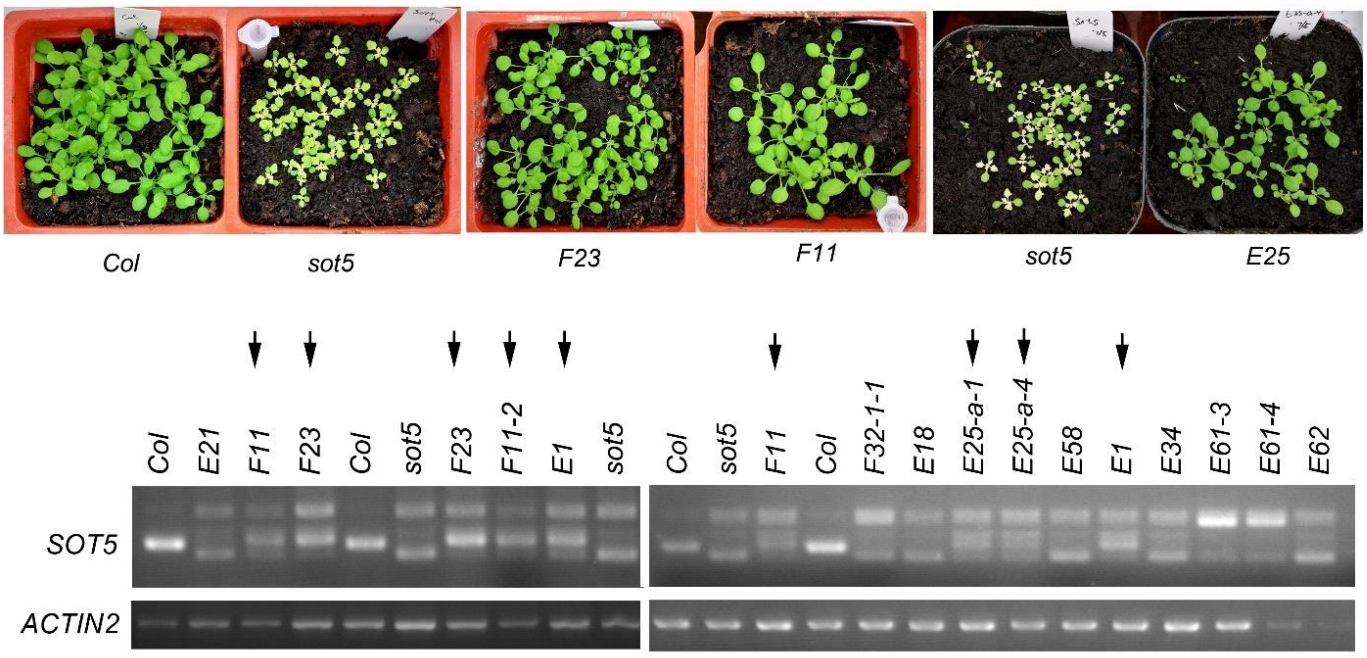

Figure S3. Phenotypes of *sot5* suppressors and their splicing patterns of *SOT5* intron 7. The upper panel shows the four suppressors lines presented the WT-like phenotype. The lower panel shows the splicing patterns of *SOT5* intron 7 in different suppressors lines. Among these suppressors, *F23, F11, E1* and *E25* (indicated by

the arrows) show a similar splicing pattern of *SOT5* intron 7.

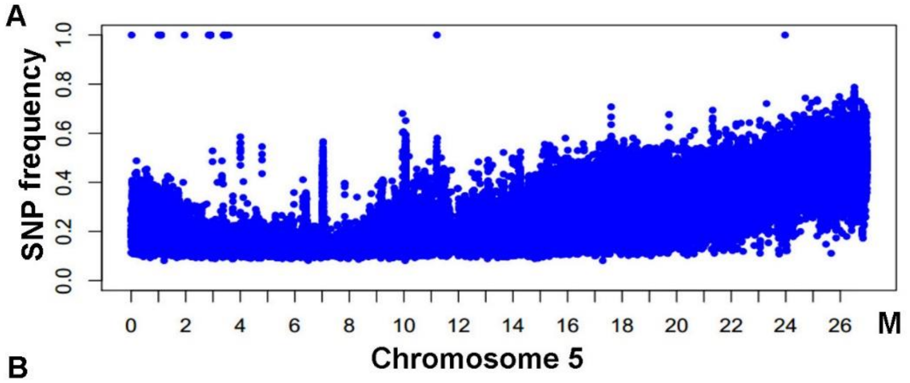

B

Gene_name	TYPE	Ratio	GO_term
AT1G30610	missense_variant	1	chloroplast;embryo development ending in seed dormancy; suspensor development
AT5G09210	missense_variant	0.909091	nucleus
AT5G15540	missense_variant	0.955556	Nucleus; sister chromatid cohesion; zinc ion binding;embryo development ending in seed dormancy;
AT5G15830	missense_variant	1	DNA binding;transcription factor activity, sequence-specific DNA binding;nucleus;
AT5G19000	missense_variant	0.931818	protein binding;nucleus;nucleus;nucleus;response to osmotic stress;protein ubiquitination
AT5G19350	stop_gained	0.958333	nucleotide binding;RNA binding;nucleus
AT5G20680	missense_variant	0.9375	endosome;Golgi apparatus;trans-Golgi network

Figure S4. Cloning the *F23* suppressor gene through the Mapping-By-Sequencing (MBS) technique. *F23* (Col background) was crossed with L*er* to generate F_2 population for gene mapping. About 80 WT-like plants with *sot5* mutation in F_2 population were selected and pooled for DNA extraction and genome re-sequencing. The frequency of SNP polymorphisms on each chromosome was then analyzed. Theoretically, *F23* mutation is closely linked with Col type SNPs. The L*er* SNP frequency close to *F23* mutation is about 0. SNPs not linked to *F23* should be half Col type and half L*er* type. (A) The frequency of SNP on chromosome 5 where *F23* mutation is located, and the blue dots represent SNP molecular markers. (B) Suppressor gene in *F23* was mapped to the interval containing six candidate genes with missense mutations and one candidate gene with the premature stop codon. And the at5g19350 which encodes the pre-mRNA splicing factor RBP45d is the most likely candidate gene.

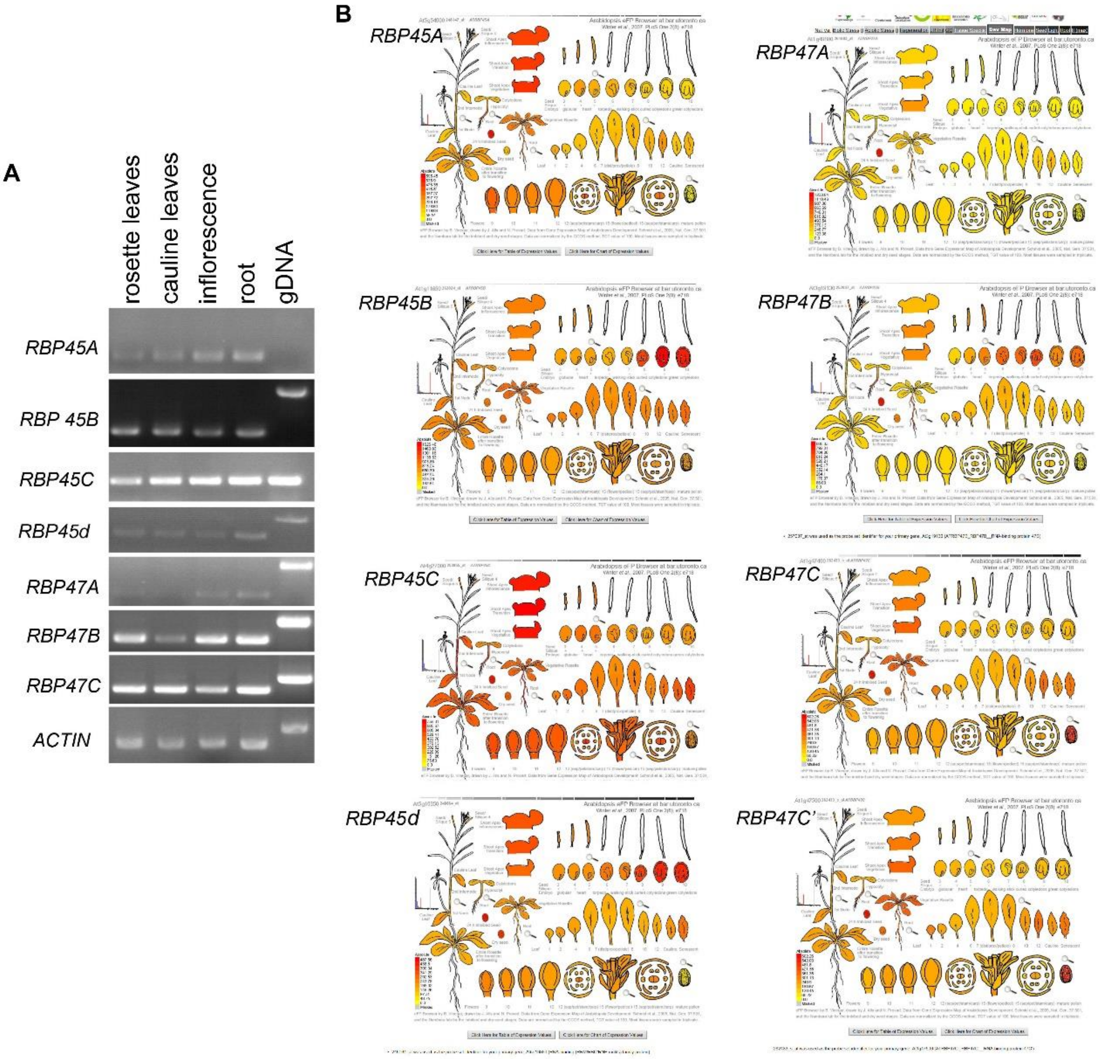

Figure S5. Expression patterns of *RBP45/47* family genes by RT-PCR and eFP browser. (A) RT-PCR analysis of *RBP45/47* gene expression in different organs. (B) eFP browser (http://bar.utoronto.ca/efp_arabidopsis/cgi-bin/efpWeb.cgi) shows expression patterns of *RBP45/47* genes in different organs.

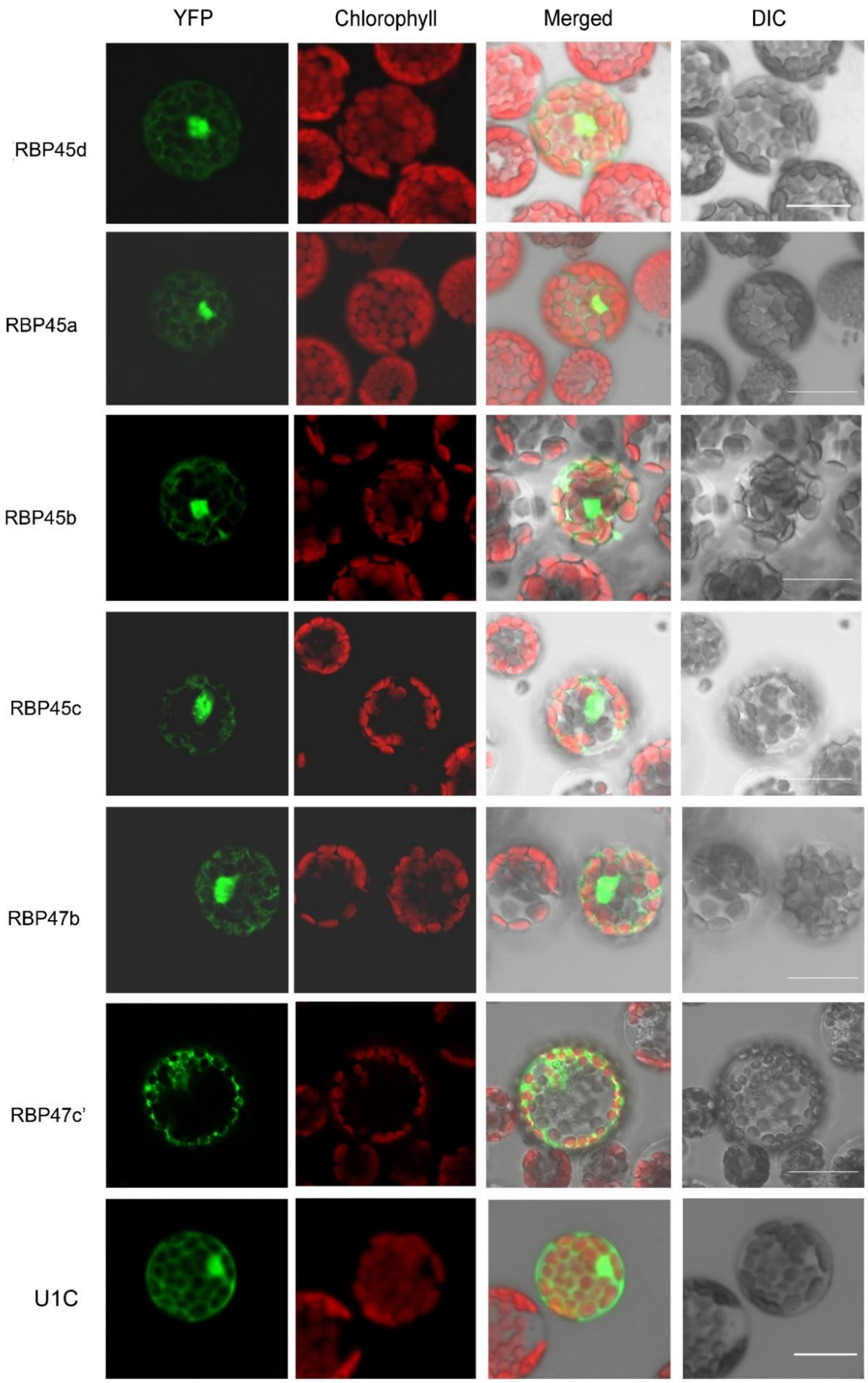

Figure S6. Subcellular localization of RBP45/47 family proteins and U1C. Confocal microscopy analysis of YFP fusion proteins transiently expressed in Arabidopsis protoplasts. The yellow fluorescence of the proteins was overlapped with chloroplast autofluorescence in merged images. DIC, Differential interference contrast microscopy. Bars = 15 μm

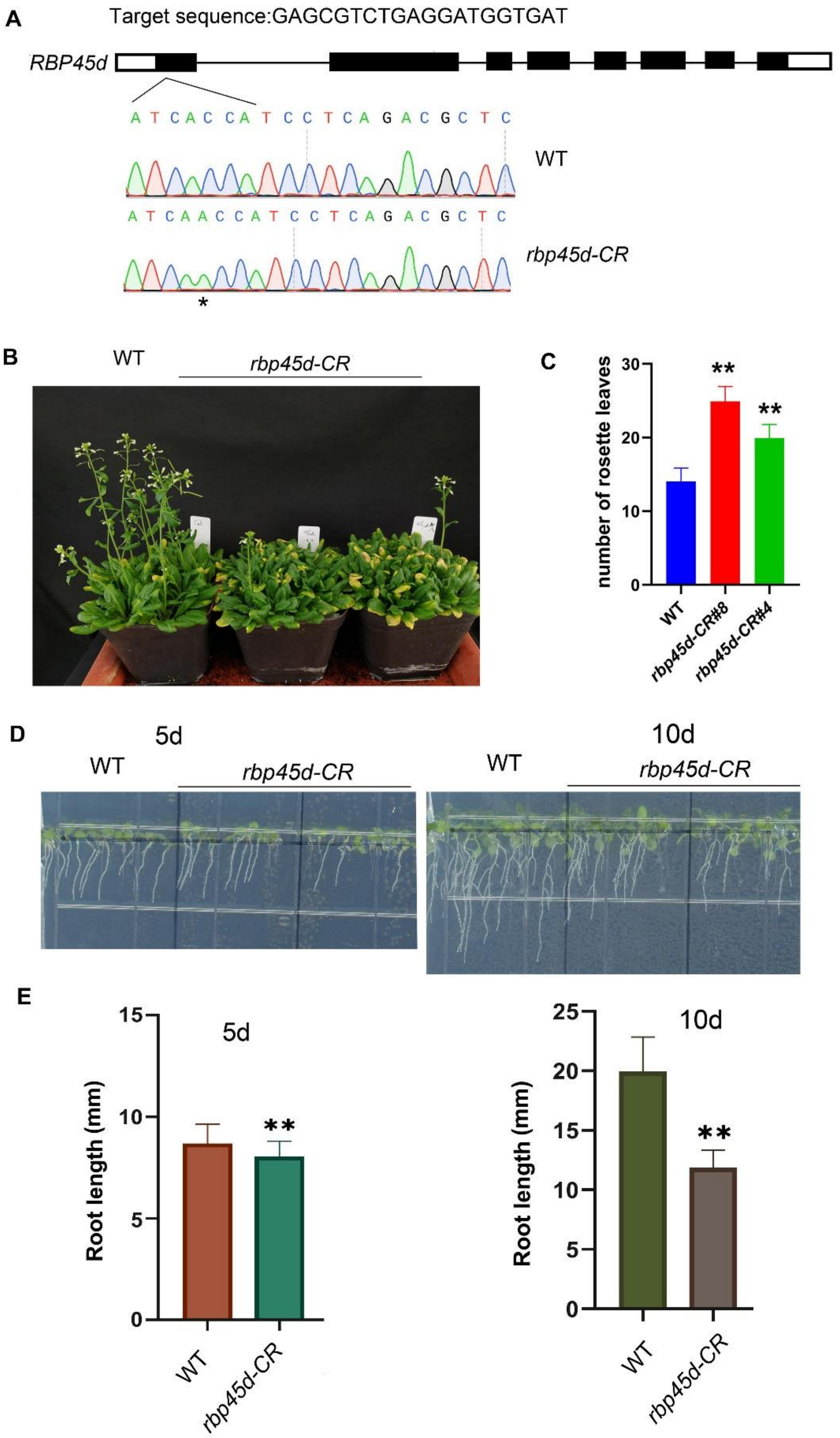

Figure S7. Phenotypes of *rbp45d-CR* plants generated by CRISPR/CAS9 technique. (A) The sgRNA sequence and the mutation site of the *rbp45d-CR* plant. * indicates the inserted A which led to frameshift of RBP45d in *rbp45d-CR* plant. (B) The phenotype of 45d-old *rbp45d-CR* plants grown under the 16h light/8h dark photoperiod. (C) The statistical analysis of *rbp45d-CR* rosette leaf number when flowering. The data are

means ± SD (n = 20). Student's *t*-test; **, $P < 0.01$. (D) Short primary root phenotype of the 5-d- and 10-d-old *rbp45d-CR* seedlings. (E) Quantification of root length shown in (D). The data shown are means ± SD (n = 20). Student's *t*-test; **, $P < 0.01$. The experiments were performed at least two times.

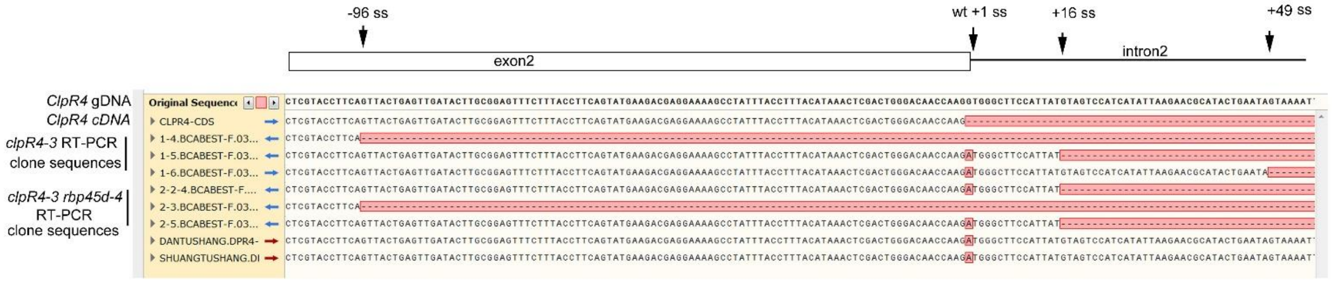

Figure S8. Sequences of the splicing products of *ClpR4* mRNA in *clpR4-3* and *clpR4-3 rbp45d-4* mutants. The DNA sequences from different clones are aligned by SnapGene software. The sequences nearing *ClpR4* intron 2 are showed.

A

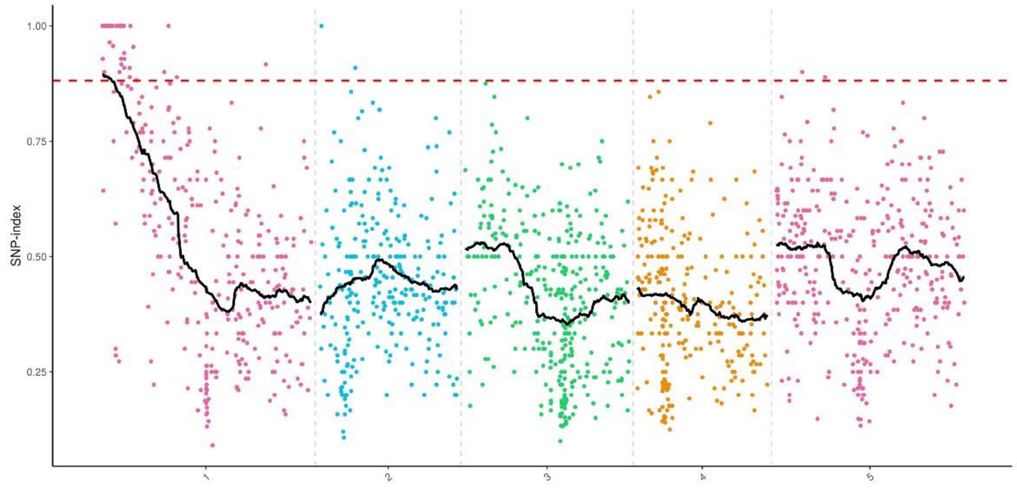

B

Locus identifier	Mutation site	Mutation position	REF	ALT	Gene description
AT1G01710	exon	1__263535	G	A	acyl-CoA thioesterase II
AT1G01860	exon	1__305176	G	A	dimethyladenosine transferase
AT1G03060	exon	1__722222	G	A	BEACH-DOMAIN HOMOLOG A1 (BCHA1) SPIRRIG (SPI)
AT1G03080	exon	1__737273	T	A	NETWORKED 1D (NET1D)
	exon	1__737282	A	G	
AT1G03290	intron	1__807767	G	A	ELKS/Rab6-interacting/CAST family protein;
AT1G03910	intron	1__997588	G	A	CACTIN (CTN)
AT1G04080	intron-splice site	1__1056104	G	A	Encodes a U1 small nuclear ribonucleoprotein (snRNP) factor involved in alternative splicing. PRP39A
AT1G04160	exon	1__1089456	T	C	Encodes a member of the type XI myosin protein family involved in root hair elongation.
	exon	1__1089471	A	T	
AT1G04180	5'-UTR	1__1106220	G	A	YUCCA 9
AT1G04445	5'-UTR	1__1207865	C	T	C2H2-like zinc finger protein
AT1G04470	exon	1__1212954	G	A	hypothetical protein (DUF810)
AT1G04650	intron	1__1297597	G	A	FIDGETIN-LIKE-1 INTERACTING PROTEIN (FLIP)
AT1G04920	exon	1__1394096	G	A	SUCROSE PHOSPHATE SYNTHASE 3F (SPS3F)

Figure S9. Cloning the *E25* suppressor gene through Mutmap technique. The BC_1 F_2 population was generated via a cross between *E25* and *sot5*. The DNA of thirty *E25* plants from the BC_1 F_2 population were extracted individually and pooled for Whole Genome Resequencing. (A) SNP index plots of five chromosomes of the *E25* mutant. Colored points indicate SNP positions and their indices. Black lines are regression lines. (B) *E25* gene was mapped in a region (~1.3 M) on Chromosome 1 which contains 15 candidate genes. Among them, at1g04080 which encodes PRP39a was the most likely candidate gene.

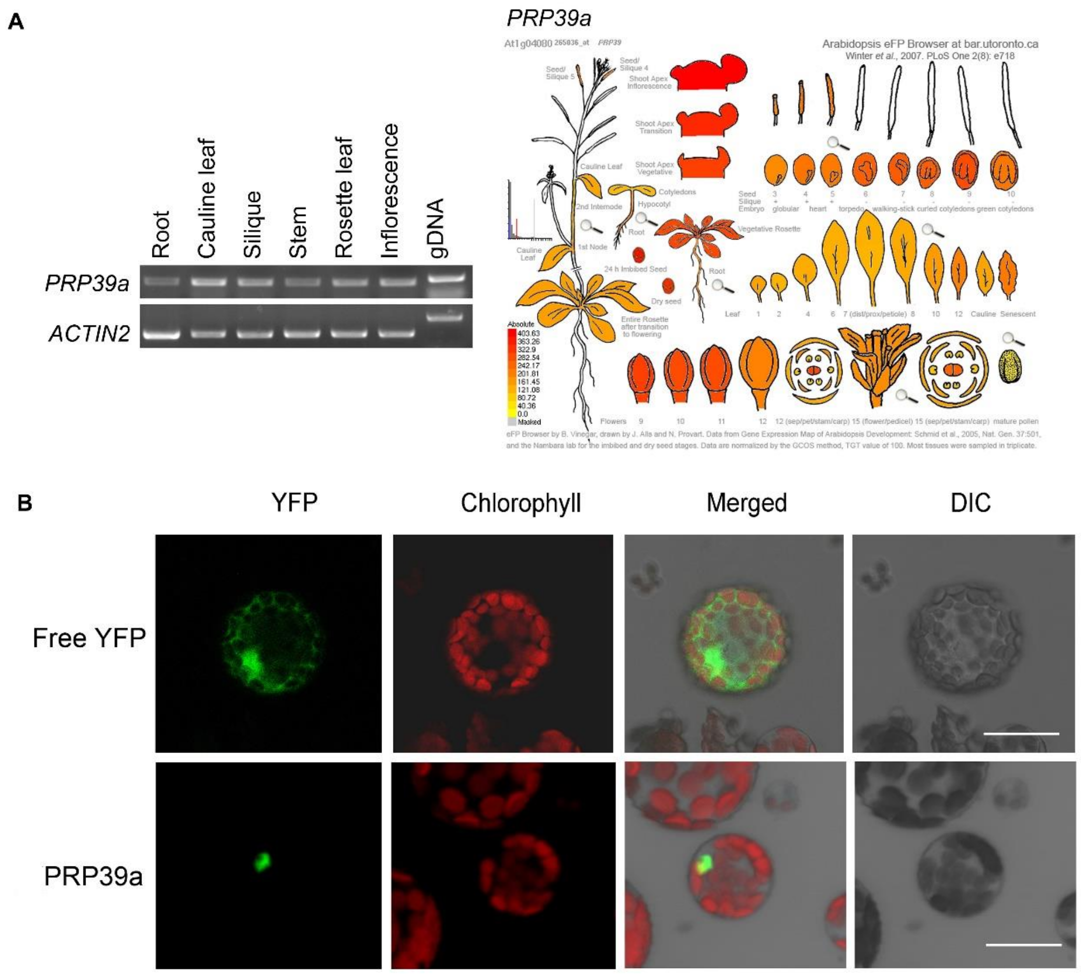

Figure S10. Expression pattern of *PRP39a* and subcellular localization of PRP39a protein. (A) RT-PCR analysis and eFP browser (http://bar.utoronto.ca/efp_arabidopsis/cgi-bin/efpWeb.cgi) show the expression pattern of *PRP39a* gene. (B) Subcellular localization of PRP39a. Microscopy analysis of the YFP fusion proteins transiently expressed in Arabidopsis protoplasts. The green fluorescence of the proteins was overlapped with chloroplast autofluorescence in merged images. DIC, Differential interference contrast microscopy. Bars = 15 μm.

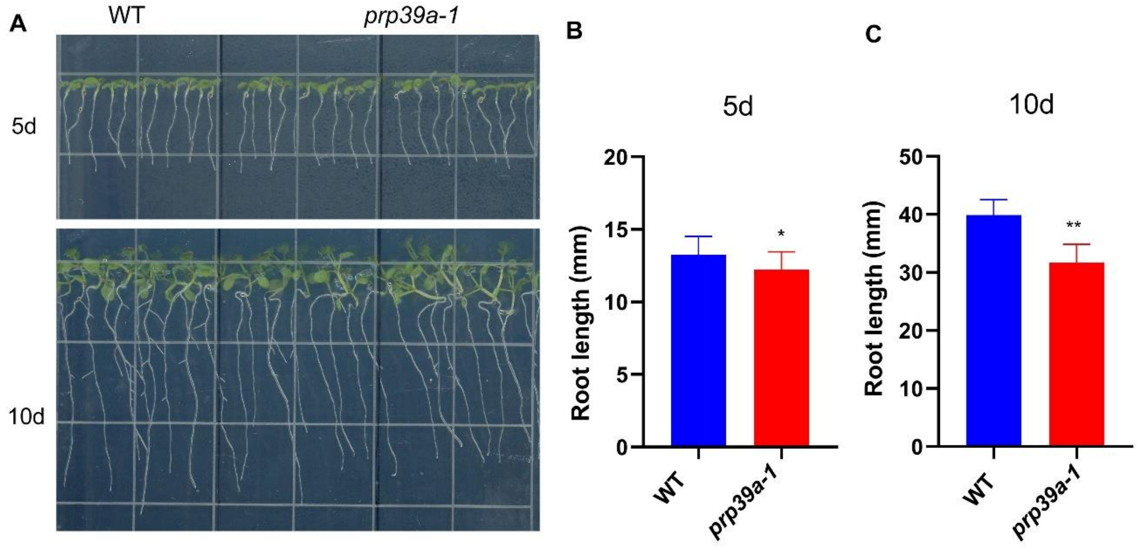

Figure S11. The *prp39a-1* mutant also exhibits the short primary root phenotype. (A) Short primary root phenotype of the 5d- and 10d-old *prp39a-1* seedlings. (B) and (C) are root length of 5d- and 10d-old seedlings. The data shown are means ± SD (n = 20). Student's *t*-test; *, $P < 0.05$; Bar = SD. The experiments were performed at least two times.
